# Advancing sustainable medication use in healthcare: a Delphi study on (de)prescribing interventions

**DOI:** 10.1136/bmjopen-2025-115383

**Published:** 2026-05-07

**Authors:** Elisabeth Marissa Smale, Jiske Lianne van der Giessen, Cathelijne Willemijne Yvonne Appels, Emiel Leegwater, Marlieke Dietz, Patricia Maria Lucia Adriana van den Bemt, Saskia Coenradie, Rudolf Bertijn Kool, Henk-Frans Kwint, Erwin Ista, Nicole Hunfeld

**Affiliations:** 1 Department of Adult Intensive Care, Erasmus MC University Medical Centre, Rotterdam, The Netherlands; 2 Reumazorg Zuid West Nederland, Roosendaal, The Netherlands; 3 Department of Pharmacy, Radboud University Medical Centre, Nijmegen, The Netherlands; 4 Department of Internal Medicine, Alrijne Hospital Leiden, Leiden, The Netherlands; 5 Department of Clinical Pharmacy, University Medical Centre Utrecht, Utrecht, the Netherlands; 6 Department of Pharmacy, Reinier de Graaf Gasthuis, Delft, The Netherlands; 7 IQ Healthcare, Scientific Institute for Quality of Healthcare, Radboud University Medical Centre, Nijmegen, The Netherlands; 8 SIR Institute for Pharmacy Practice and Policy, Leiden, The Netherlands; 9 Department of Pediatric Surgery, Intensive Care Unit, Erasmus MC Sophia Kinderziekenhuis, Rotterdam, The Netherlands; 10 Department of Internal Medicine, Nursing Science, Erasmus MC University Medical Centre, Rotterdam, The Netherlands; 11 Department of Hospital Pharmacy, Erasmus MC University Medical Centre, Rotterdam, The Netherlands

**Keywords:** Pharmacists, Hospitals, Physicians, Implementation Science, Prescriptions, Delphi Technique

## Abstract

**Abstract:**

**Objective:**

To identify and prioritise the most appropriate (de)prescribing interventions in inpatient and outpatient hospital care to advance environmentally sustainable healthcare.

**Design:**

A modified RAND Delphi study.

**Setting:**

Inpatient and outpatient hospital care in the Netherlands.

**Participants:**

The Delphi panel consisted of 63 participants, comprising 36 physicians and 27 pharmacists working in Dutch hospitals.

**Primary and secondary outcome measures:**

Consensus on the appropriateness of (de)prescribing interventions for frequently used medications in inpatient and outpatient hospital care to advance environmentally sustainable healthcare and the prioritisation of interventions per care setting (inpatient/outpatient) and intervention type (deprescribing/sustainable dosage form), culminating in a top 20.

**Results:**

51 (de)prescribing interventions were identified for 18 medication classes, for which consensus on appropriateness was reached for 42 (82%). The top 20 highest ranked interventions were identified, starting with switching from intravenous to oral administration of paracetamol, stopping chronically used proton pump inhibitors without indication and initiating antibiotics orally in case of good bioavailability.

**Conclusions:**

Most (de)prescribing interventions were considered appropriate for advancing sustainable medication use, highlighting support for their potential implementation to reduce the environmental burden of healthcare.

**STRENGTHS AND LIMITATIONS OF THIS STUDY**

- The study addresses a key knowledge gap by prioritising (de)prescribing strategies aimed at environmental sustainability, ensuring real-world feasibility and effectiveness through a Delphi panel of healthcare professionals responsible for implementing these interventions.
- It systematically combines real-world data, literature research, expert opinion and Delphi methodology to provide evidence-based recommendations aligned with current healthcare practices.
- Limitations include focusing on frequently prescribed medication classes, which may have excluded subgroups with significant environmental impact, and the inability to directly assess the environmental impact of interventions due to a lack of data.

## Introduction

 The healthcare sector has a high environmental impact, accounting for 4.4% of greenhouse gas emissions worldwide, with medication representing a major contributor.^1^ The environmental burden of medication arises across its entire life cycle, spanning material extraction, production, use and disposal. Pharmaceutical manufacturing is associated with substantial energy consumption and greenhouse gas emissions,^2^ requiring vast amounts of (non-recyclable) packaging materials^3^ and relying on extensive global supply chains.^4^ In addition, medication residues may accumulate in the environment, after use, by patients or due to improper disposal, posing a risk to aquatic life and drinking water quality.^5^ Given this considerable environmental footprint, a more critical and balanced approach to medication use is needed, in which therapeutic benefits are carefully weighed against the potential harms to both patients and planetary health.

Optimising medication use and reducing low-value prescriptions offer important opportunities to improve clinical outcomes and patient safety while improving environmental sustainability through the reduction of the footprint associated with manufacturing and distributing these medications.^6 7^ This approach is grounded in the understanding that reducing unnecessary medication use not only improves patient care but also minimises the environmental impact of pharmaceuticals.^8^ Medications for which the potential harms outweigh their therapeutic benefits are considered potentially inappropriate.^9 10^ Studies show that up to 92% of patients may receive potentially inappropriate medications, depending on the population, clinical context and drug class.^11^ For instance, chronic acid-suppression therapy has been reported to be unnecessary in up to 88% of patients.^12^ Another opportunity to substantially lower the environmental footprint of medication is to choose the most environmentally friendly dosage form when equally effective. While evidence on environmentally sustainable prescribing remains limited,^13^ promising examples have emerged. For instance, dry-powder and soft mist inhalers contain 200–920 g CO_2_-equivalent less per inhaler compared with pressurised metered-dose inhalers,^14^ and administering paracetamol orally instead of intravenously reduces the carbon footprint up to 16 times.^15 16^ Sustainable prescribing therefore encompasses both clinical and environmental optimisations, ensuring that patients receive the most effective and environmentally responsible treatment at the optimal dose while avoiding unnecessary or harmful medications.^17^

Although both patients and healthcare providers are willing to consider environmental sustainability in their medication choices, evidence on how sustainability can be integrated into prescribing practice is lacking.^18 19^ For instance, 71% of global clinical guidelines do not include deprescribing recommendations, and to date, no studies have quantified the environmental impact of deprescribing.^20 21^ However, implementing (de)prescribing interventions could substantially reduce the environmental impact of medication use and, by extension, healthcare. Therefore, the national programme ‘Greening Healthcare Together’ was initiated in the Netherlands to implement (de)prescribing interventions for improving environmental sustainability in inpatient and outpatient care.^22^ However, it remains unclear which medications are most appropriate to target and to what extent sustainable (de)prescribing strategies are feasible. The current study, therefore, aims to identify and prioritise the most appropriate (de)prescribing interventions to advance environmentally sustainable healthcare.

## Methods

### Design

A modified three-round RAND Delphi study was conducted with physicians and pharmacists employed in Dutch hospitals from April to July 2025 to identify and prioritise appropriate (de)prescribing interventions to advance environmental sustainability.^23^ The study protocol is shared in the supplementary materials (online supplemental information 1) and follows the "Delphi studies in social and health sciences-recommendations for an interdisciplinary standardized reporting" (DELPHISTAR) reporting guidelines, provided in online supplemental information 2.^24^ No consulting with regard to methods took place besides the involved authors.

### Identifying relevant (de)prescribing interventions

To identify potential (de)prescribing interventions, the most frequently prescribed medication classes in the inpatient and outpatient settings of Dutch hospitals were identified, based on the hypothesis that targeting high-volume prescriptions would yield a greater environmental impact. For inpatient care, the top 20 most frequently purchased medications were determined from data of four hospitals, including two university medical centres and two teaching hospitals, over the period February 2024–February 2025. The top 20 most frequently used medications by outpatients were identified from insurance claims in 2024, because the Dutch healthcare system is based on compulsory health insurance.^25^ Medications were categorised into classes based on Anatomical Therapeutic Chemical (ATC) classification levels 2 (ie, therapeutic main group), 3 (ie, pharmacological subgroup) and, if relevant, level 4 (ie, chemical subgroup).^26^ Inclusion criteria for both inpatient and outpatient settings were: (1) containing an active pharmaceutical ingredient and (2) the potential to adjust or switch the prescription in line with Dutch guidelines on when medications with the same active substance, strength and formulation can be responsibly modified or substituted, taking into account clinical equivalence, patient safety and conditions under which switching is appropriate.^27^ An additional inclusion criterion for the outpatient setting was that a reasonable share of these prescriptions related to hospital care, which was assessed based on expert opinion.

All medication classes were matched to (de)prescribing interventions and options for switching to the most sustainable dosage form. This was based on (inter)national (de)prescribing guidelines and literature, specifically through MEDLINE, Embase and Web of Science (online supplemental information 3). Inclusion criteria were based on the intervention, context and outcomes. Interventions included deprescribing (eg, lower-dose prescribing, tapering and withdrawal), reducing overprescribing (eg, targeting inappropriate medicines), dosing strategies (eg, increased dosing intervals and dose banding), eco-directed prescribing (eg, green pharmaceuticals and low-carbon options) and sustainable dosage forms (eg, tablets vs liquids and oral vs parenteral). The context included medications initiated in both inpatient and outpatient hospital care. Outcome criteria were based on dose reduction (eg, mg or percentage of active ingredient) or environmental impact (eg, greenhouse gas emissions for eco-directed prescribing and sustainable dosage forms). Exclusion related to study characteristics, including reviews, commentaries, current state descriptions (eg, waste levels, concentrations in water and qualitative research on the current state). Quality assessments were conducted by the first author and reviewed by the expert panel consisting of (hospital) pharmacists (in training), medical specialists (in training) and (de-)implementation experts. The expert panel was also invited to include additional interventions based on their knowledge and experience. The identified deprescribing interventions and options for switching to the most sustainable dosage forms per medication (class) formed the input for the Delphi study.

### Study population

The study population consisted of pharmacists, including hospital pharmacists, outpatient pharmacists and residents, and physicians, such as medical specialists and residents, working in either an inpatient or outpatient setting, in Dutch hospitals, because together they are responsible for prescribing practices for inpatients and outpatients. Participants were recruited through an open call distributed via newsletters of professional associations of hospital pharmacists and medical specialists, as well as through social media (eg, LinkedIn). Maximum variation was sought to ensure that the Delphi panel reflected diverse perspectives across professional roles, career stages and clinical domains.

To account for a minimum 70% response rate per round, initially 30 participants per stakeholder group (eg, 30 physicians and 30 pharmacists) were recruited to ensure that at least 20 participants would complete all rounds of the Delphi study.^28^ Participants were invited to each Delphi round separately, and participation in a previous round was not a requirement to join subsequent rounds.

### Delphi study

A Delphi study was conducted to systematically gather the judgement of different expert groups, for example, physicians and pharmacists, to identify consensus on the most appropriate (de)prescribing intervention to advance environmental sustainability of healthcare. The Delphi study was performed online through LimeSurvey (LimeSurvey, 2025). Panellists received email invitations with a personal, anonymous login code and were sent reminders after 1 week and 2 days before survey closure. Each Delphi round remained open for a minimum of 2 weeks, with extensions applied if the participation rate was below 70%. As an incentive, trees were planted for each participant, with one tree for completing the first survey, two for the second and three for the third.

The Delphi study consisted of three Delphi rounds in total. In all communications, sustainable prescribing was defined as ‘the clinical and environmental optimisation of treatment to ensure that patients receive the most effective and environmentally responsible treatment at the optimal dose while avoiding unnecessary or harmful medications’ in relation to deprescribing (eg, reducing unnecessary medication use by not starting, lowering the dosage, tapering or stopping medications) and/or switching to more sustainable administration forms (eg, choosing the most sustainable variant of a medication related to the route of administration and form). After an introduction on study objectives, informed consent and general personal characteristics, including occupation, medical specialty and type of hospital of employment, participants were requested to rate interventions per setting (outpatient vs inpatient) and intervention type (deprescribing vs switching to the most sustainable dosage form) (online supplemental information 4). Participants were requested to rate all the interventions on appropriateness for implementation in Dutch hospitals to improve environmental sustainability, with specific consideration of (1) the effectiveness and safety of the treatment remain guaranteed; (2) the environmental impact of the treatment is reduced, for example, by reduced dosage and/or less packaging material and (3) the initiative can be implemented in Dutch hospitals. Additionally, other factors for evaluation were pointed out, specifically the level of scientific evidence or practical experience demonstrating that the initiative is appropriate for reducing environmental impact and how well the interventions fit into existing care pathways and/or their ease of implementation. All interventions were substantiated with the type of evidence, including a link to the corresponding research.

### Appropriateness of (de)prescribing interventions

#### Data collection

The first two Delphi rounds evaluated the appropriateness of potential (de)prescribing interventions within each medication class per setting (outpatient vs inpatient) and intervention type (deprescribing vs switching to the most sustainable dosage form). Interventions were singularly assessed based on appropriateness, stating ‘this (intervention) is appropriate for implementation in Dutch hospitals to reduce environmental impact’. Participants could rate their agreement with each statement based on a 7-point Likert scale (1=‘strongly disagree’ to 7=‘strongly agree’). During the first round, comprehensiveness of the (de)prescribing interventions was evaluated by asking participants if they knew any other (de)prescribing interventions. Every page contained open text space to leave remarks on specific interventions. In the first Delphi round, each section concluded with an open-ended question asking for suggestions on any missing interventions. These additional suggestions were then used to refine and inform the interventions tested in the subsequent rounds.

#### Data analysis

Consensus on appropriateness was determined using median scores in combination with feedback and Disagreement Index, calculated as the interpercentile range (IPR) adjusted for symmetry (IPRAS), following the RAND/UCLA (University of California, Los Angeles) Delphi methodology.^23^ Since a 7-point Likert scale was used, a Disagreement Index>0.7 indicated disagreement (IPR≥IPRAS), whereas ≤0.7 indicated consensus. Interventions with a median score of 5–7 were classified as appropriate and included, while those with a median score of 1–3 were classified as inappropriate and excluded. In all other cases, sustainability initiatives were reassessed in a subsequent Delphi round.

Suggestions for additional (de)prescribing interventions and remarks were assessed qualitatively and categorised in ‘new interventions to be added’, ‘feedback to interventions’, ‘feedback to Delphi process’ and ‘suggestions outside of study scope’. Two authors (EMS and JLvdG) reviewed these categories. Comments in the first two categories were clustered and used to inform subsequent Delphi rounds. Interventions with intermediate median scores, disagreement and/or qualitative feedback were reassessed in the second Delphi round, in which median scores were fed back to participants. If the open text space contained questions, specialist justification was incorporated to address these in the subsequent Delphi round to all participants. Interventions with consensus and no qualitative feedback in the first round were not presented in the second round.

### Prioritising (de)prescribing interventions

#### Data collection

The last Delphi round was used to rank the (de)prescribing interventions in order to prioritise these to advance the environmental sustainability of Dutch hospital care. Participants were asked to rank the interventions by compiling four separate top 5 lists: one for outpatient deprescribing, one for inpatient deprescribing, one for outpatient switch to the most sustainable dosage forms and one for inpatient switch to the most sustainable dosage forms.

#### Data analysis

Through their ranks, different interventions were prioritised per category and overall. The score for each intervention was calculated by weighting the number of times it was assigned a given rank, with higher ranks receiving greater weight. To enable comparison across categories with different numbers of interventions, the score was corrected for the maximum number of possible ranks within that category. Formally, the score per intervention was calculated as:


$$Score=\sum_{r=1}^{N}nr*\left(N-r+1\right)$$

where *r* represents the assigned rank to the intervention, nr the number of participants who were assigned rank *r* and *N* the number of interventions within the category. Higher rank sum scores indicate a stronger overall preference for the intervention, with greater consensus among panellists. Conversely, lower rank sum scores suggest less agreement or a lower prioritisation of the intervention. Interventions with the highest scores per category were compared, resulting in a top 20 of prioritised (de)prescribing interventions for hospital care.

### Patient and public involvement

Patients and/or the public were not involved in the design, conduct, reporting or dissemination plans of this research.

## Results

### Identifying relevant (de)prescribing interventions

Based on prescribing data and predefined inclusion criteria, 18 therapeutic groups were identified as targets for (de)prescribing interventions in Dutch hospital care (online supplemental information 5), resulting in 43 (de)prescribing interventions. Based on participants’ feedback in the first Delphi round, additional (de)prescribing interventions were added (n=8), and interventions were revised (n=4). Collectively, 51 (de)prescribing interventions were identified across 27 medication classes (online supplemental information 6).

Deprescribing interventions constituted the majority (n=28, 55%) and were equally distributed across inpatient and outpatient settings (both n=14, 28%), while sustainable dosage forms (n=23) were primarily applied in inpatient settings (n=14, 28%) compared with outpatient settings (n=9, 18%). Almost all main ATC groups were targeted by the (de)prescribing interventions, including alimentary tract and metabolism (n=9, 18%), blood and blood-forming organs (n=7, 14%), cardiovascular (CV) system (n=9, 18%), genitourinary system and sex hormones (n=1, 2%), anti-infectives for systemic use (n=6, 12%), antineoplastic and immunomodulating agents (n=1, 2%), nervous system (n=11, 22%), respiratory system (n=3, 6%) and sensory organs (n=1, 2%).

### Delphi panel

**Figure 1:**
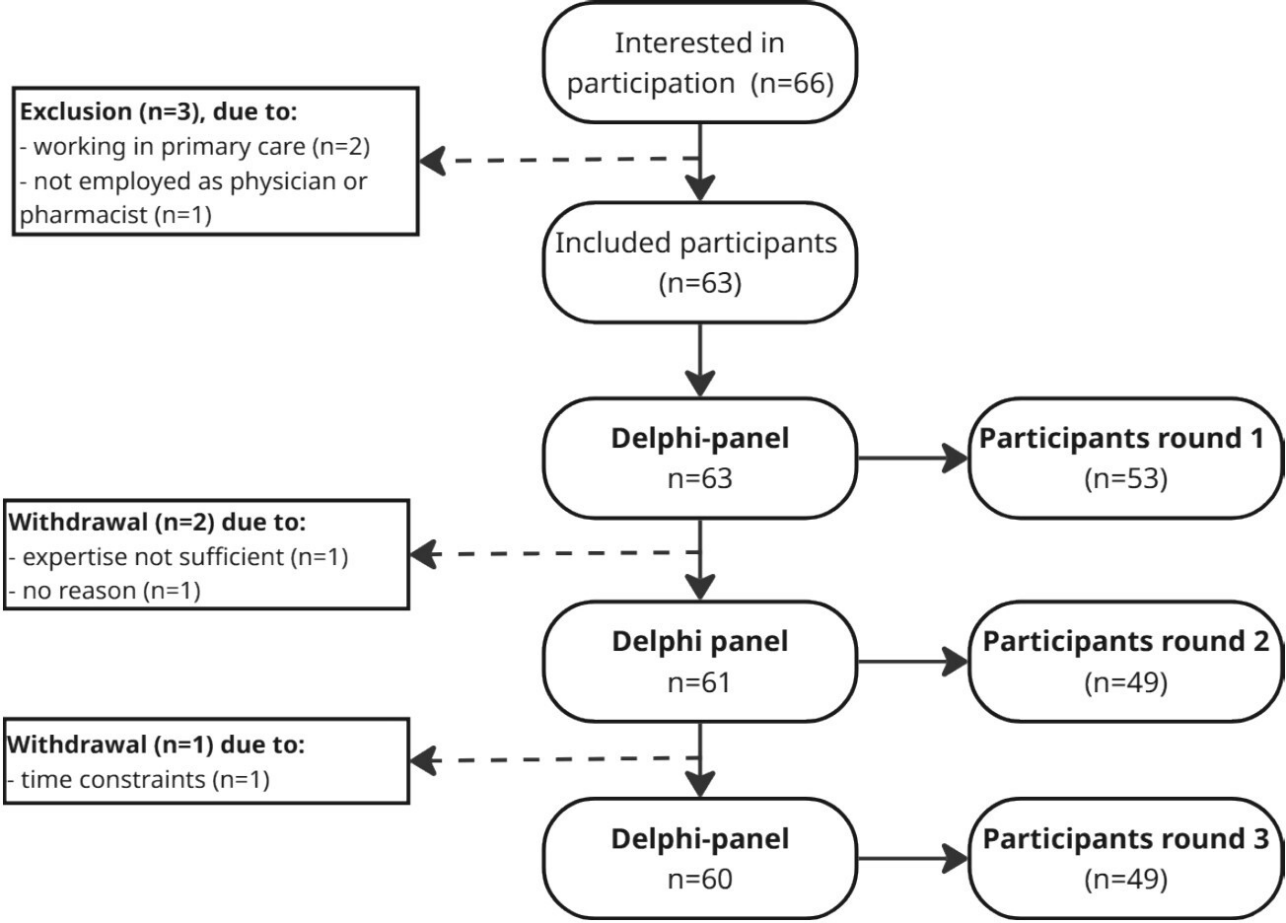
Flow diagram of participants of the Delphi study.

In total, 66 individuals expressed interest in participation, resulting in a Delphi panel of 63 participants who met the inclusion criteria (figure 1). The Delphi panel consisted of 36 physicians, including 27 medical specialists and 9 residents, and 27 pharmacists, including 17 hospital pharmacists, 6 outpatient pharmacists and 4 hospital pharmacy residents, employed in 35 different hospitals (table 1). Most participants were employed in teaching hospitals (n=28, 44%), followed by general hospitals (n=16, 25%) and university medical centres (n=16, 25%), with smaller numbers from independent treatment centres (n=2, 3%) and a specialised hospital (n=1, 2%).

**Table 1:** Panel characteristics (n=63)

Profession	Medical specialties	Panel, n (%)	Round 1, n (%)	Round 2, n (%)	Round 3, n (%)
Physicians	Cardiology	1 (2)	1 (2)	1 (2)	1 (2)
	Dermatology	3 (5)	2 (4)	3 (6)	2 (4)
	Surgery	1 (2)	1 (2)	1 (2)	1 (2)
	Emergency medicine	1 (2)	1 (2)	1 (2)	1 (2)
	Geriatrics	1 (2)	0 (0)	0 (0)	0 (0)
	Gynaecology	2 (3)	2 (4)	1 (2)	2 (4)
	Internal medicine	11 (17)	11 (21)	10 (20)	10 (20)
	Intensive care	1 (2)	1 (2)	1 (2)	1 (2)
	Ophthalmology	1 (2)	1 (2)	0 (0)	1 (2)
	Otorhinolaryngology	1 (2)	1 (2)	1 (2)	1 (2)
	Paediatrics	1 (2)	1 (2)	1 (2)	0 (0)
	Pulmonology	3 (5)	3 (6)	3 (6)	3 (6)
	Rheumatology	9 (14)	6 (11)	3 (6)	4 (8)
	***Total***	**36 (57)**	**31** (**58**)	**26** (**53**)	**27** (**55**)
Pharmacists	Hospital pharmacy	21 (33)	18 (34)*	18 (37)*	18 (37)*
	Outpatient pharmacy	6 (10)	5 (9)*	6 (12)*	7 (14) *
	***Total***	**27 (43)**	**22 (43)**	**23 (47)**	**22 (45)**
**Total**		**63 (100)**	**53 (100)**	**49 (100)**	**49 (100)**

The totals of each (sub) group are displayed in bold.

* Some pharmacists were employed in both the outpatient pharmacy and hospital pharmacy.

The first round had 53 respondents (participation rate 84%), of whom 31 were physicians (58%) and 22 (42%) were pharmacists. Two participants (one medical specialist and one hospital pharmacist) withdrew during the first round due to self-reported lack of expertise on this topic and for no other reason. The second round had 49 respondents (participation rate 80%), of whom 26 (53%) were physicians and 23 (47%) pharmacists. After the second round, another participant (medical specialist) withdrew due to time constraints. The final round had 49 respondents again (participation rate 82%), with 27 physicians (55%) and 22 pharmacists (45%). In total, 296 trees were planted to thank the panel for their participation.

### Evaluating the appropriateness of (de)prescribing interventions

In the first Delphi round, consensus on appropriateness was reached for 30 of 43 (70%) (de)prescribing interventions (online supplemental information 6, table 1). The highest-scoring interventions were ‘stopping chronically used proton pump inhibitors (PPIs) without indication’ and ‘discontinuing statins in advanced disease’ (median score of 7). The Disagreement Index indicated agreement for all interventions (≤0.7). For eight interventions, consensus was not reached; all received a median score of 5 (online supplemental information 6, table 2). These interventions concerned deprescribing in the inpatient (n=3) and outpatient settings (n=5).

**Table 2:** Overview of identified (de)prescribing interventions for inpatients and their appropriateness (A)

Therapeutic group		Medication class	Type	Prescribing intervention	Reference	A
A02	Drugs for acid-related disorders	PPIs	D	Avoid routine PPI initiation in patients at low risk	^34^	+
			D	Stop chronic use of PPIs without an indication		+
			S	Prescribe oral pantoprazole instead of intravenous administration	^35 36^	+
A03	Drugs for functional disorders	Propulsives	S	Prescribe *enteral* metoclopramide instead of intravenous administration		+
A04	Antiemetics and antinauseants	5-HT3 antagonists	S	Prescribe oral ondansetron or granisetron instead of intravenous administration		+
A10	Drugs used in diabetes	Blood glucose lowering drugs	D	Reconcile/taper dosing based on HbA1c and glucose	^37^	-
B01	Antithrombotic agents	Antithrombotic agents	D	Actively monitor stop dates of combination therapy during admission	^38^	+
B03	Antianaemic preparations	Iron preparations	S	Administer oral iron tablets instead of liquid iron, *every other day instead of daily*	^39^	+
C03	Diuretics	Loop diuretics	S	Administer loop diuretics as a bolus injection instead of an infusion	^40^	+
			S	Prescribe oral loop diuretics instead of intravenous administration in non-acute situations	^35 36^	+
			D	Do not initiate beta-blockers post-MI in patients with normal cardiac function	^41^	+
C10	Lipid-modifying agents	HMG CoA reductase inhibitors	D	Discontinue statins in patients with advanced disease	^42^	+
J01	Antibacterials for systemic use	Various	D	Add a stop date when initiating antibiotic treatment (course)	Expert opinion	+
			D	Add a stop date when initiating prophylactic antibiotics		+
			S	Administer antibiotics as a bolus injection instead of an infusion	^43^	+
			S	Initiate oral antibiotics with good bioavailability	^44 45^	+
J01		Cephalosporins	S	For cephalosporin prophylaxis, prefer once-daily dosing where possible	Delphi	+
M01	Anti-inflammatory drugs	NSAIDs	D	Replace NSAIDs with adequate paracetamol dosing	Expert opinion	+
N01	Anaesthetics	Other general	S	Replace propofol 1% with propofol 2%	Delphi	−
N02	Analgesics	Opioids	S	Prescribe oral morphine instead of intravenous morphine	^35 36^	+
			S	Prescribe oral morphine instead of oxycodone	^46^	+
			D	Reduce opioid prescriptions at discharge by standardised pain plans	^47 48^	+
			D	Reduce opioid prescriptions at discharge by including a stop date		+
			D	Stepwise pain management (no routine opioid prescribing) after caesarean section	^49^	+
		Paracetamol	S	Prescribe oral paracetamol instead of intravenous administration (*paracetamol challenge*)	^16^	+
N05	Psycholeptics	Benzodiazepines	D	Avoid initiating benzodiazepines; use non-pharmacological interventions	^50 51^	−
			D	Discontinuation of benzodiazepines in patients receiving geriatric care	^52 53^	+
			S	Prescribe oral lorazepam instead of intravenous administration as premedication	^35 36^	+

Adjustments made after the first Delphi round are shown in italics.

A: appropriateness; + indicates yes and − indicates no.

D, deprescribing intervention; HbA1C, Hemoglobin A1c; HMG CoA, 3-hydroxy-3-methylglutaryl-coenzyme A; 5-HT3, 5-hydroxytryptamine type 3 receptor; MI, myocardial infarction; NSAIDs, non-steroidal anti-inflammatory drugs; PPIs, proton pump inhibitors; S, switch to the most sustainable dosage form.

**Figure 2:**
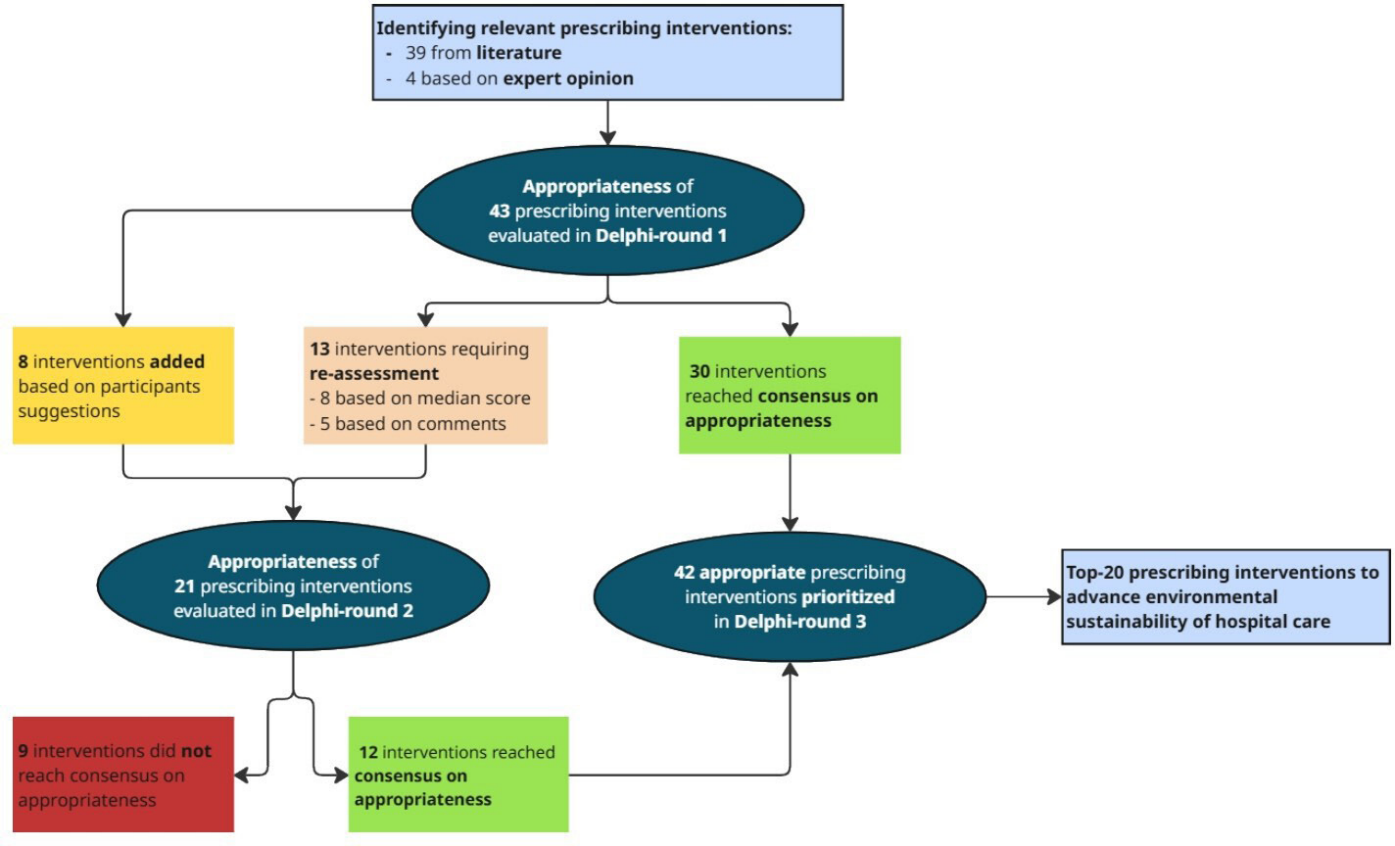
Flow diagram of (de)prescribing interventions assessed in the Delphi study.

In the second Delphi round, 21 (de)prescribing interventions were (re)assessed, including 8 interventions without consensus carried forward from round one, 5 interventions that required reassessment due to adjustments after approval in the first round and 8 interventions newly added in the second round (figure 2). Consensus was reached for 12 interventions (57%) (online supplemental information 6, tables2^4^).

**Table 3:** Overview of identified (de)prescribing interventions for outpatients and their appropriateness (A)

Therapeutic group		Medication class	Type	Prescribing intervention	Reference	A
A02	Drugs for acid-related disorders	PPIs	D	Stop chronic use of PPIs without indication	^34^	+
A06	Drugs for constipation	Laxatives	D	Regularly evaluate laxative use and discontinue when opioids are stopped	Delphi	+
A10	Drugs used in diabetes	Blood glucose-lowering drugs	D	Adjust therapy (eg, stop combination or reduce dose) if hypoglycaemia risk is present or targets are achieved	^37^	−
B01	Antithrombotic agents	Antithrombotic agents	D	Monitor stop dates of combination therapy and share indication/duration with primary care providers	^54^	+
			D	Short-term DAPT after drug-eluting stenting in patients with ACS	^55^	+
			D	Stop antiplatelets for primary CV prevention *in patients aged >70 years*	^56^	−
B03	Antianaemic preparations	Iron preparations	S	Administer oral iron tablets instead of liquid iron, *every other day instead of daily*	^39^	+
		Erythropoietic growth factors	S	Administer epoetin beta weekly instead of 3–7 times per week in patients with cancer	^57^	+
C02	Anti-hypertensives	Various	D	Taper preventive antihypertensives in patients with low CV risk	^58^	−
C03	Diuretics	Thiazides	D	Review chronic diuretic use to taper/stop if clinically appropriate	^59^	−
C07	Beta-blocking agents	Beta-blocking agents	D	Stop beta-blockers post-MI in patients with normal cardiac function (LVEF>50%)	^41^	+
C09	Agents acting on the RA system	ACEi/ARB	D	Re-evaluate ACEi+ARB combination therapy	Delphi	−
C10	Lipid-modifying agents	HMG CoA reductase inhibitors	D	Taper statins in patients with low CV risk	^58^	+
G04	Urologicals	Alpha-adrenoreceptor antagonists	D	Stop alpha-1 blockers after 6 months in men with LUTS	^56^	+
J01	Antibacterials for systemic use	Various	S	Evaluate the possibility of oral switch in OPAT	Delphi	+
L04	Immunosuppressants	Other immunosuppressants	S	Switch high-dose methotrexate from subcutaneous to split-dose oral administration	Delphi	−
M01	Anti-inflammatory drugs	NSAIDs	D	Replace NSAIDs with adequate paracetamol dosing	Expert opinion	+
M02	Anti-inflammatory and antirheumatic products	NSAIDs topical	S	Reduce use of topical NSAID gels and switch to oral therapy where possible	Delphi	+
N02	Analgesics	Opioids	D	Reduce opioid use by communicating intended treatment duration with primary providers	^47 48^	+
R03	Drugs for obstructive airway diseases	Various	S	Initiate dry powder inhalers instead of metered-dose inhalers where possible	60 62	+
		Various	S	Switch from metered-dose inhalers to dry powder inhalers instead, where possible	60 62	+
		Various	D	Re-evaluate inhaled corticosteroids after 1 year with COPD	^56^	+
S01	Ophthalmologicals	Various	S	Use multidose eye drops instead of unit-dose flacons to reduce plastic waste	Delphi	+

Adjustments made after the first Delphi round are shown in italics.

A: appropriateness; + indicates yes and − indicates no.

ACEi, angiotensin-converting enzyme inhibitors; ACS, Acute Coronary Syndrome; ARB, angiotensin receptor blocker; COPD, chronic obstructive pulmonary disease; CV, cardiovascular; D, deprescribing intervention; DAPT, double antiplatelet therapy; HMG CoA, 3-hydroxy-3-methylglutaryl-coenzyme A; LUTS, lower urinary tract symptoms; LVEF, left ventricular ejection fraction; MI, myocardial infarction; NSAIDs, non-steroidal anti-inflammatory drugs; OPAT, outpatient parenteral antimicrobial therapy; PPIs, proton pump inhibitors; RA, renin angiotensin; S, switch to most sustainable dosage form.

**Table 4:** Top 20 prioritised (de)prescribing interventions in hospital care

Overall rank	Cat. rank	Medication class	Setting	Type	Prescribing intervention	RSW
1	1	Paracetamol	Inpatient	S	Prescribe oral paracetamol instead of intravenous paracetamol (*paracetamol challenge*)	429
2	1	PPIs	Outpatient	D	Stop chronic use of PPIs without indication	351
3	2	Various	Inpatient	S	Initiate oral antibiotics with good bioavailability	342
4	1	Opioids	Inpatient	D	Reduce opioid prescriptions at discharge by standardised pain plans	293
5	2	PPIs	Inpatient	D	Stop chronic use of PPIs without indication	287
6	3	PPIs	Inpatient	D	Avoid routine PPI initiation in patients at low risk	283
7	3	Various	Inpatient	S	Administer antibiotics as a bolus injection instead of an infusion	281
8	2	Opioids	Outpatient	D	Reduce opioid use by communicating intended treatment duration with primary providers	262
9	4	Opioids	Inpatient	D	Reduce opioid prescriptions at discharge by including a stop date	261
10	4	Propulsives	Inpatient	S	Prescribe *enteral* metoclopramide instead of intravenous administration	257
11	5	Various	Inpatient	D	Add a stop date when initiating antibiotic treatment (course)	245
12	6	HMG CoA reductase inhibitors	Inpatient	D	Discontinue statins in patients with advanced disease	243
13	5	Opioids	Inpatient	S	Prescribe oral morphine instead of oxycodone	232
14	3	Antithrombotic agents	Outpatient	D	Monitor stop dates of combination therapy and share indication/duration with primary care providers	231
15	6	Iron preparations	Inpatient	S	Administer oral iron tablets instead of liquid iron, *every other day instead of daily*	230
16	7	Opioids	Inpatient	S	Prescribe oral morphine instead of intravenous morphine	229
17	1	Various	Outpatient	S	Initiate dry powder inhalers instead of metered-dose inhalers where possible	227
18	2	Various	Outpatient	S	Switch from metered-dose inhalers to dry powder inhalers instead, where possible	220
19	4	NSAIDs	Outpatient	D	Replace NSAIDs with adequate paracetamol dosing	208
20	5	Various	Outpatient	D	Re-evaluate inhaled corticosteroids after 1 year with COPD	193

Adjustments made after the first Delphi round are shown in italics.

A higher RSW indicates the greatest consensus and higher prioritisation of the intervention.

Cat, Category, defined by type of setting (inpatient/outpatient) and type of intervention (deprescribing/switch to most sustainable dosage forms); COPD, chronic obstructive pulmonary disease; D, deprescribing intervention; HMG CoA, 3-hydroxy-3-methylglutaryl-coenzyme A; NSAIDs, non-steroidal anti-inflammatory drugs; PPIs, proton pump inhibitors; RSW, rank of sum weighing; S, sustainable prescribing intervention.

After two Delphi rounds, consensus was reached on the appropriateness of 42 of 51 (82%) (de)prescribing interventions (figure 2). Table 2 displays the evaluated (de)prescribing and their appropriateness for inpatients, while table 3 summarises the interventions for outpatients. Of the 30 deprescribing interventions, 22 (67%) were deemed appropriate, comprising 10 of 16 (63%) outpatient and 12 of 14 (86%) inpatient interventions. Most interventions on switching to the most sustainable dosage form (20 of 22, 91%) were considered appropriate, with consensus reached for 13 of 14 (93%) inpatient interventions and 7 of 8 (88%) outpatient interventions.

### Prioritising (de)prescribing interventions

Of the top 20 prioritised interventions (table 4), the majority applied to inpatient settings (n=13, 65%), comprising both deprescribing (n=6, 30%) and switching to the most sustainable dosage form (n=7, 35%). In comparison, outpatient setting interventions (n=7, 35%) were mainly deprescribing (n=5, 25%), with only a minority focused on sustainable dosage forms (n=2, 10%). The most frequently represented medication classes were analgesics (n=6, 30%), mainly opioids (n=5) and paracetamol (n=1), followed by PPIs (n=3, 15%), antibacterials for systemic use (n=3, 15%) and inhalation medication (n=3, 15%). Other medication classes represented were iron preparations (n=1, 5%), statins (n=1, 5%), NSAIDs (n=1, 5%), antithrombotic agents (n=1, 5%) and propulsives (n=1, 5%).

## Discussion

To our knowledge, this is the first study to identify and prioritise (de)prescribing interventions aimed at reducing the environmental impact of healthcare. Using the Delphi methodology, the study achieved consensus among physicians and pharmacists on the most appropriate interventions. The identified interventions focused on deprescribing and sustainable prescribing in both inpatient and outpatient care, spanning a wide range of medication classes, including analgesics, antibiotics, anticoagulants, antiemetics, anti-inflammatory drugs, CV medicines, PPIs and inhalation medication. Since these prioritised, evidence-based interventions can help reduce the environmental impact of medications, eight of these (combined) interventions will be (de-)implemented and evaluated across Dutch hospitals as part of the national programme ‘Greening Healthcare Together’ (online supplemental information 7).

This study prioritised environmentally sustainable prescribing interventions that offer both environmental gains and clinical co-benefits, which is in line with previous research on the preferences of healthcare professionals.^29^ Medications like antibiotics, which have a relatively large environmental footprint,^30^ are prime targets for sustainability efforts, particularly because of the co-benefits of addressing antimicrobial resistance and potentially reduced hospitalisation duration.^7^ Similarly, consistent with previous findings from Choosing Wisely, prescribing interventions for opioids and antithrombotic therapies were rated highly, as they can improve patient safety while reducing low-value care.^31^

Previous research also pointed out that targeting high-volume care was perceived as a motivator for sustainability interventions in healthcare.^29^ While the highest-rated interventions involved paracetamol, the most widely used medication globally, high prescription volume alone did not determine prioritisation. This is evident from the inclusion of several antithrombotic therapies, which, despite their relatively low usage, were prioritised due to their significant clinical benefits.^12 32^ Beyond prescribing volume and clinical value, familiarity also appeared to influence prioritisation. In the current study, this was evident from the strong support for interventions that are already widely implemented, such as the intravenous to oral switch of paracetamol.^16^ These findings indicate that widespread communication on sustainability practices may facilitate the acceptance and uptake of sustainability initiatives within healthcare.

A key strength of this study is its systematic approach to identifying and prioritising (de)prescribing interventions using real-world data, literature research, expert opinion and the Delphi methodology. This approach ensures that the recommendations are evidence-based but also aligned with current healthcare practices, as the Delphi panel encompassed the healthcare professionals responsible for executing and implementing them. Still, several limitations should be noted. First, the study focused on the most frequently prescribed medication classes, which, while hypothesised to have the largest environmental impact, may have overlooked specific subgroups with significant benefits. As an additional consequence, a broad panel was required, which sometimes lacked expertise in certain areas; for example, some participants provided feedback that they felt uncomfortable rating specific interventions. Stratification by medical specialty was not feasible due to small sample sizes, but in the second Delphi round, specialist justification was incorporated to address such gaps. Another limitation was the lack of environmental impact data, which prevented comparisons of interventions based solely on environmental sustainability. However, this also strengthened the findings of the study, as appropriateness was defined beyond just environmental factors, incorporating other key aspects such as feasibility and scalability. Finally, while broad recruitment interventions were employed, selection bias may have been introduced due to the over-representation of rheumatologists and healthcare professionals with an interest in environmental sustainability. Despite these limitations, the broad consensus reached helped to ensure that the findings are robust and relevant.

Evidence on the environmental impact of (de)prescribing interventions remains limited,^20 21^ underscoring the need for studies that quantify environmental outcomes. Linking prescription data with environmental impact assessments could provide a better understanding of the contribution of prescribing practices to sustainable healthcare. This will be further evaluated for the prioritised interventions within Dutch hospitals as part of the Greening Healthcare Together programme.^22^ Another key priority is determining how to effectively implement these (de)prescribing interventions in clinical practice. Successful implementation will require close collaboration between prescribers, pharmacists and policymakers, as well as integration into existing clinical guidelines to support green behavioural change among healthcare professionals. However, the factors that influence healthcare professionals’ adoption of these behaviours remain unclear. Although behavioural change in deprescribing has been studied,^33^ the role of environmental sustainability in this context is still understudied. Future research should therefore focus on developing and evaluating strategies to achieve sustainable (de)prescribing practices at scale. Equally important is incorporating the patient perspective, which was not directly addressed in this study. Evidence from a large-scale evaluation among Dutch medicine users indicates that patients generally prioritise environmental sustainability over convenience and cost.^19^ However, these attitudes are heterogeneous, and many patients lack specific knowledge about the environmental impact of medicines. To foster patient-centred care that balances sustainability goals with patient needs and preferences, future studies should actively assess and integrate patient perspectives. Systematically embedding environmental sustainability into prescribing in this way can ensure interventions are acceptable and effective, ultimately contributing to both improved patient care and planetary health.

## Conclusions

This Delphi study identified and prioritised (de)prescribing interventions to reduce the environmental impact of healthcare. Most prescribing interventions were considered appropriate by pharmacists and physicians, highlighting support for their potential implementation. Specifically, (de)prescribing interventions that offer co-benefits, such as improved patient safety and more appropriate care alongside reduced environmental impacts, obtained priority. These results form the foundation for the national Greening Healthcare Together programme, which aims to implement (de)prescribing interventions in Dutch hospitals.

## Supplementary material

10.1136/bmjopen-2025-115383 online supplemental file 1

## Data Availability

Data are available upon reasonable request.
