# Supplementary material for: Advancing sustainable medication use in healthcare: a Delphi study on (de)prescribing interventions
Source: BMJ Open. 2026 May 7;16(5):e115383. doi: 10.1136/bmjopen-2025-115383 (PMC13157796; doi:10.1136/bmjopen-2025-115383)
Supplement: online supplemental file 1 [file bmjopen-16-5-s001.docx]

**Advancing sustainable medication use in health care:**

a Delphi-study on (de)prescribing interventions

Supplementary Material

Table of contents

[**Supplement 1: Study protocol** 3](#_Toc215560523)

[Summary 4](#_Toc215560524)

[Introduction and rationale 5](#_Toc215560525)

[Objective 6](#_Toc215560526)

[Study type 6](#_Toc215560527)

[Study population 7](#_Toc215560528)

[Methods 8](#_Toc215560529)

[Incidental findings 10](#_Toc215560530)

[Statistical analysis 11](#_Toc215560531)

[Ethical considerations 12](#_Toc215560532)

[Handling and storage of data / images / sound recordings / photos / film recordings 13](#_Toc215560533)

[Handling and storage of human material 14](#_Toc215560534)

[Exchange, sharing or transfer of data and/or human material and/or images 14](#_Toc215560535)

[Amendments 14](#_Toc215560536)

[End of study report 14](#_Toc215560537)

[Publication 14](#_Toc215560538)

[References 15](#_Toc215560539)

[**Supplement 2: DELPHISTAR Reporting guidelines for Delphi-studies** 17](#_Toc215560540)

[**Supplement 3: Literature Search** 20](#_Toc215560541)

[**Supplement 4: Questionnaire** 22](#_Toc215560542)

[Round 1/2 22](#_Toc215560543)

[Round 3 27](#_Toc215560544)

[**Supplement 5: Selected medication classes for prescribing interventions** 30](#_Toc215560545)

[**Supplement 6: Appropriateness and prioritization of (de)prescribing interventions** 31](#_Toc215560546)

[**Supplement 7: selected (de)prescribing interventions** 38](#_Toc215560547)

## **Supplement 1: Study protocol**

​ List of abbreviations and relevant definitions*

**ATC** Anatomical Therapeutic Chemical

**CTA** Clinical Trial Agreement

**De novo biobank** A new data, human material or imaging collection

**DMP** Data Management Plan

**DPIA** Data Protection Impact Assessment

**DTA** Data Transfer Agreement

**Exception consent** Form Care for data Template, in Dutch: Formulier uitzondering toestemming

**GCP** Good Clinical Practice

**GDPR** General Data Protection Regulation

**GHG** greenhouse gas

**IC** Informed Consent

**IFU** Instruction For Use

**MTA** Material Transfer Agreement

**NWTC** Non-WMO Review Committee; in Dutch: Niet WMO Toetsingscommissie

**UAVG** Dutch Act on Implementation of the General Data Protection Regulation; in Dutch: Uitvoeringswet Algemene Verordening Gegevensbescherming

**WMO** Medical Research Involving Human Subjects Act, in Dutch: Wet Medisch-wetenschappelijk Onderzoek met Mensen

### Summary

**Rationale**Upscaling  strategies, like deprescribing and switching to the most sustainable dosage form, can help to improve the environmental sustainability of medication use. However, it remains unclear which medication (classes) are appropriate to target with sustainable strategies in Dutch in- and outpatient hospital care.

**Objective(s)**

To identify which medication (classes) are appropriate to target with deprescribing and switching to sustainable dosage forms to improve the environmental sustainability of Dutch in- and outpatient hospital care.

**Study type**Modified RAND Delphi-Study

**Study population**

The study will involve a diverse sample of physicians and pharmacists working in Dutch hospitals. Inclusion criterion is being an adult healthcare professional employed in a hospital and exclusion criterion for physicians is not being familiar with prescribing the selected medications.

**Methods** 
The most frequently used medication will be determined based on the number of users in in- and outpatient care in the Netherlands. Inclusion will be based on (I) containing an active pharmaceutical ingredient (API), (II) regular prescriptions in hospital care, and (III) possibility of modifying the prescription based on Dutch guidelines. Medication will be categorized in classes by Anatomical Therapeutic Chemical (ATC) Classification level 4 (e.g., pharmacological subgroup), with individual assessment for those with differing indications. Strategies for deprescribing and switching to sustainable dosage forms will be identified for the twenty most frequently used medication (classes) in in- and outpatient care based on literature, guidelines for sustainable alternatives, and expert opinion.

A Delphi-study with a panel of medical specialists and hospital pharmacists will be used to rate the appropriateness of the potential strategies for sustainable medication use per selected medication (class) on a 7-point Likert scale (1 = “strongly disagree”, 7 = “strongly agree”). Median appropriateness ratings and the disagreement index (DI) will be calculated to classify strategies as appropriate (median scores 5 - 7), uncertain, or inappropriate (median scores 1 – 3). The last Delphi-round will be used to rank the strategies to identify which medication (classes) are (most) appropriate to target; selecting eight strategies (two relating to inpatient care and two relating to outpatient care) to be implemented in Dutch hospitals.

**Burden and risks** 
The study population only concerns healthcare professionals. The only burden of filling out online surveys to reach consensus regards the time investment.

**Recruitment and consent**  
The study population encompasses healthcare professionals who receive information about the study purpose through e-mail. At the first page of the survey participants are requested permission to use their data.

### Introduction and rationale

The healthcare sector has an alarming environmental impact, accounting for 4.4% of the greenhouse gas (GHG) emissions worldwide, with medication serving as a major contributor to this impact (1-4). Primarily, production of medication has a substantial carbon footprint (5, 6), but the use of medication gives rise to more environmental stressors. Medication residues may accumulate in the environment after use by patients or due to improper disposal, inducing severe ecological disturbances (7, 8). Moreover, the non-recyclable packaging materials of medication drive resource consumption and waste generation (9). Given the considerable environmental footprint of medication use, a more critical and balanced approach to medication use is needed — one that carefully weighs the therapeutic benefits against the potential harms to both patients and the broader perspective of planetary health.

Suboptimal use of medication undermines patient well-being and unnecessarily drives the environmental burden of medication. Prescribed medication of which the potential harm outweighs the intended therapeutic benefits is referred to as inappropriate (10). In the UK, estimates suggest that up to 10% of the primary prescriptions are inappropriate, including medication that is no longer needed, not matching the needs of individual patients, or for which a better alternative is available (11). This definition could be extended as inappropriate medication also encompasses unnecessary high doses or medication for which equally effective therapeutic options are available with a lower environmental impact (12), relating to the route of administration (e.g. oral administration versus parenteral administration (13)) and dosage form (e.g. dry-powder inhalers versus pressured metered-dose inhalers (14)). Sustainable medication use thus means that patients receive the most sustainable medication or dosage form in the optimal dosage and do not receive any unnecessary medication.

Sustainable strategies informed by circularity principles (15), like reducing and rethinking medication use, can help to improve environmental sustainability. To date, several strategies for deprescribing (16) and switching to the most sustainable dosage form (13, 14) have been implemented to improve treatment and/or reduce environmental burden of medication. Upscaling these strategies to other medications frequently used in hospital care can further reduce the environmental impact of medication use. What remains unclear is which medication (classes) are appropriate to target, and to what extent the strategies are appropriate to implement in the Dutch healthcare system. The current study is therefore set out to identify which medication (classes) are appropriate to target with deprescribing and switching to sustainable dosage forms to improve the environmental sustainability of Dutch in- and outpatient hospital care.

### Objective

To identify which medication (classes) are appropriate to target with deprescribing and switching to sustainable dosage forms to improve the environmental sustainability of Dutch in- and outpatient hospital care.

### Study type

**3.1. Study type**

**​​☐​** Retrospective

**​​☒​** Prospective

**​​☐​** Combination Retrospective/Prospective

**3.2 Single center / Multicenter**

**​​☐​** Single center

**​​☒​** Multicenter

**3.3 Check all the applicable boxes**

**​​☐​** Medical records (re-use of data from healthcare, including AI)

**​​☐​** Case report

**​​☐​** Re-use data from research

**​​☐​** Evaluations of quality of healthcare (retrospective)

**​​☐​** Research with additional use of residual material from regular healthcare

**​​☐​** Research with re-use of human material from research or existing biobank

**​​☐​** De novo biobank

**​​☐​** Phase IV research

**​​☐​** Healthcare evaluation research (prospective)

**​​☐​** Research with medical devices

**​​☐​** Research with In Vitro Diagnostic Tests

**​​☒​** Other research, describe: **Delphi-study**

### Study population

**4.1. Study population**

**​☒​** Adults (16 years and older)

**​​☐​** Minors (younger than 16 years)

**​​☐​** Incapacitated adults (16 years and older)

**​​☐​** Incapacitated minors (younger than 16 years) 

**4.2. Population (base)**

The population includes physicians and pharmacists working in Dutch hospitals. By means of purposive sampling a diverse sample based on function, type of hospital (e.g., academic, top clinical or peripheral) and field of expertise will be compiled.

**4.3. Inclusion criteria**

An adult (healthcare) professional being employed as a physician or pharmacist in a Dutch hospital.

**4.4. Exclusion criteria**

Physicians who are not familiar with prescribing the selected medications, which will be based on their field of expertise.

**4.5. Sample size calculation**

A sample size of 20 per individual stakeholder group is required to ensure reliable results in a Delphi-study(17). Furthermore, to maintain rigor, it is important that the response rate in each Delphi round remains above 70%(18). Therefore, we aim to recruit 30 participants per stakeholder group (e.g., 30 physicians and 30 pharmacists) to ensure that at least 20 participants per stakeholder group will complete all rounds of the Delphi-study.

### Methods

**5.1. Research methods**

A modified RAND Delphi-study (19) will be conducted to identify which medication (classes) are appropriate to target with deprescribing and switching to sustainable dosage forms to improve the environmental sustainability of Dutch in- and outpatient hospital care.

The Delphi-study consists of idea generation (e.g., selection of most frequently used medication; identifying deprescribing strategies and sustainable dosage forms per medication (class), followed by evaluation of their appropriateness to implement in Dutch hospitals (Figure 1).


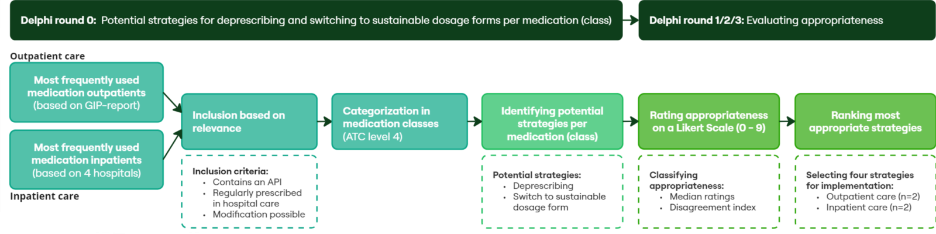


**Figure 1: Schematic overview of study**

GIP: Genees- en hulpmiddelen Informatie Project Data registry; API: active pharmaceutical ingredient; ATC: Anatomical Therapeutic Chemical.

Delphi round 0: Potential strategies per medication (class)

This study will focus on medication (classes) that are widely used throughout the Netherlands, to maximize the environmental impact. For outpatient care, medications will be extracted from the ‘Genees- en hulpmiddelen Informatie Project’ (GIP) report ‘medication with most users’. For inpatient care, the twenty most frequently administered medications will be extracted from the purchasing data of four hospitals.

Inclusion is based on (I) containing an active pharmaceutical ingredient (API), (II) regular prescriptions in hospital care, and (III) possibility of modifying the prescription according to the Dutch guideline ‘Responsible switching’ and exclusion of antibiotics due to the strict national guidelines (20, 21). Medication will be categorized into classes based on Anatomical Therapeutic Chemical (ATC) Classification level 4 (e.g., pharmacological subgroup) (22). To ensure the survey remains concise and manageable for participants, we will limit the list to the top 20 most frequently used medication classes per care setting.  In classes where the indications vary significantly, individual medications will be assessed separately to account for their specific characteristics. All medication (classes) will be matched to deprescribing strategies and options for switching to sustainable dosage forms, based on literature, (de)prescribing guidelines, and expert opinion of a panel consisting of (hospital) pharmacists (in training), medical specialists (in training) and (de)implementation experts. The identified deprescribing strategies and options for switching to sustainable dosage forms per medication (class) will be the input for the subsequent rounds of the Delphi-study.

Delphi-rounds 1/2/3: Evaluating the appropriateness of sustainable strategies per medication (class)

A panel of medical specialists and hospital pharmacists will be requested to rate the appropriateness of each potential sustainable strategy per medication class to improve environmental sustainability of Dutch hospital care. The Delphi-study will be conducted digitally through Limesurvey. Each potential strategy for a certain medication (class) will be scored on a 7-point Likert scale (1 = “strongly disagree”, 7 = “strongly agree”), see F1 ‘B. Delphi rounds 1-3’. Through open text space panelists can substantiate their answer and/or give suggestions for improvement.

Panelists will be recruited through an open call, for instance spread in the newsletter of the professional associations of hospital pharmacists and medical specialists and through social media and using snowball sampling techniques to obtain a maximum variation sample. Each Delphi-round will be available to participants for two weeks, or longer when a participation rate of 70% has not yet been achieved. Participants will receive a reminder after every week of no response. The Delphi-rounds will continue until round three or will stop sooner if consensus is reached on all strategies (see 5.4. Analysis). The last Delphi-round will be used to rank the strategies to identify which medication (classes) are (most) appropriate to target with deprescribing and/or switching to sustainable dosage forms to enhance the environmental sustainability of Dutch in- and outpatient hospital care; selecting eight strategies (at least two relating to inpatient care and two relating to outpatient care) that will be implemented in Dutch hospitals.

**5.2. Standard clinical care versus extra for research**

The study is non-therapeutic; the research population consists of healthcare professionals who are requested to participate in (several) online surveys to reach consensus on appropriate sustainability strategies for medication to implement in Dutch hospitals.

**5.3. Burden and risks**

The study population only concerns healthcare professionals. The only burden of filling out online surveys to reach consensus regards the time investment.

**5.4. Medical device(s) / In vitro diagnostic tests**

NA

### Incidental findings

**6.1. Chance of incidental findings** 
Is there a chance of incidental findings?

**​​☐​** Yes

**​​☒​** No

**6.2. Procedures** 
NA

### Statistical analysis

**7.1. Main study parameters/endpoints** 
Medication (classes) appropriate to target deprescribing and switching to sustainable dosage forms to improve the environmental sustainability of Dutch in- and outpatient hospital care.

**7.2. Secondary study parameters/endpoints**

- To identify the top-20 medication (classes) with the most users in the Netherlands for in- and outpatient hospital care, as well as potential strategies to enhance environmental sustainability.
- The appropriateness of targeting medication (classes) with strategies to improve the environmental sustainability of medication use in Dutch hospital care.
- Selection of the eight most appropriate strategies for medication (at least two relating to inpatient care and two relating to outpatient care) that will be implemented in Dutch hospitals.

**7.3. Other study parameters** 
NA

**7.4. Analysis**

Based on the median scores on the appropriateness of using a strategy for a certain medication(class), strategies will be ranked on a 7 -point Likert Scale of agreement (1 = “strongly disagree”, 7 = “strongly agree”). Strategies with a median score between 5 – 7 will be considered appropriate and therefore be included, while strategies with a median score between 1 – 3 will be considered inappropriate and therefore be excluded (19). In all other cases, sustainability initiatives will be reassessed in a subsequent Delphi-round.

The RAND disagreement index (DI) will be calculated for each statement to assess panel disagreement by comparing the 30th–70th Interpercentile Range (IPR) to the Interpercentile Range Adjusted for Symmetry (IPRAS) (19). Strategies with DI ≥ 1 (IPR ≥ IPRAS) will be classified as uncertain, regardless of the median panel rating.

### Ethical considerations

**8.1. Regulation statement** 
The study will be conducted according to the principles of the Declaration of Helsinki (October 2024, see for the most recent version: [www.wma.net](http://www.wma.net/)) and Gedragscode Gezondheidsonderzoek 2022.

**8.2. Informed consent**

Will the subjects be asked for informed consent?

**​​☐​** Yes *(Upload Participant Information Letter and Informed Consent)*

**​​**☐**​** No, consent already given in previous study *(Upload* *Participant Information Letter and Informed Consent previous study)*

**​​☐​** No, this research will be performed under the exception consent *(Upload form Care for data Template, in Dutch: Formulier uitzondering toestemming)*

**​​☒​** Other (e.g., partly, indirectly): **prior to filling out the survey, information about the study will be shared and permission to use data will be requested to participating healthcare professionals.**

**8.3. Recruitment and informed consent procedures** 
Panelists will be recruited through an open call, for instance spread in the newsletter of the societies of hospital pharmacists and medical specialists and through social media and using snowball sampling techniques to obtain a maximum variation sample.

The study population encompasses healthcare professionals who receive information about the study purpose prior to the survey and/or through e-mail (see E1E2 ‘Information and consent form’). At the first page of the survey participants are requested permission to use their data. Per participant, trees will be planted as an incentive for participation.  

**8.4. Exception consent** 
NA

### Handling and storage of data / images / sound recordings / photos / film recordings

**9.1. Data / images / sound recordings / photos / film recordings** 
Mostly quantitative data will be collected through Limesurvey and will be documented as Microsoft Office compatible files in a secured Microsoft Teams environment, only accessible for the program team.  

**9.2. Privacy protection**

Data will be handled confidentially in line with the EU General Data Protection Regulation and the Dutch Act on Implementation of the General Data Protection Regulation. Contact details obtained from the open survey will be separately handled from the data, and the data will be pseudonymized using a code. The key-file will be secured and only accessible for involved members of the program team, which is safeguarded by the project leader. Contact details will be deleted after study completion.  

**9.3. Handling and storage of data** 
Data will be pseudonymized by Limesurvey (data management system). The key-file will be secured and only accessible for involved members of the program team, which is safeguarded by the project leader. Contact details will be deleted after study completion, other data will be kept 10 years after they are collected, in line with Erasmus MC guidelines.

**9.4. Handling and storage of images / sound recordings / photos / film recordings** 
NA

**9.5. Approval of access to data / images / sound recordings / photos / film recordings** 
After publication, metadata will be made available through the Dataverse NL repository. Approval of access to data is managed by the project leader and/or PI of the program.

### Handling and storage of human material

**10.1. Human material**

**10.2.** **Check all the boxes which are applicable to the human material origin:**

NA 
**10.3 Handling and storage of human material**

NA

- 1. **Biobank**

NA 
**10.5 Approval** **of access to human material** 
NA

### Exchange, sharing or transfer of data and/or human material and/or images

NA 

### Amendments

NA

End of study report 
Within one year after the end of the study a final study report will be submitted with the results of the study, including any publications/abstracts of the study.

### Publication

Do you have the intention to submit the study results in a manuscript for publication in a journal:

​​☒​ Yes

​​☐​ No, *please motivate*

The study will be submitted for publication no later than 2025 and the selection of strategies will be made available on our website samendezorgvergroenen.nl

### References

1. Lenzen M, Malik A, Li M, Fry J, Weisz H, Pichler P-P, et al. The environmental footprint of health care: a global assessment. The Lancet Planetary Health. 2020;4(7):e271-e9.

2. Steenmeijer MA, Rodrigues JFD, Zijp MC, Waaijers-van der Loop SL. The environmental impact of the Dutch health-care sector beyond climate change: an input&#x2013;output analysis. The Lancet Planetary Health. 2022;6(12):e949-e57.

3. Tennison I, Roschnik S, Ashby B, Boyd R, Hamilton I, Oreszczyn T, et al. Health care's response to climate change: a carbon footprint assessment of the NHS in England. The Lancet Planetary Health. 2021;5(2):e84-e92.

4. Lau I, Burdorf A, Hesseling S, Wijk L, Tauber M, Hunfeld N. The carbon footprint of a Dutch academic hospital—using a hybrid assessment method to identify driving activities and departments. Frontiers in Public Health. 2024;12.

5. De Soete W, Dewulf J, Cappuyns P, Van der Vorst G, Heirman B, Aelterman W, et al. Exergetic sustainability assessment of batch versus continuous wet granulation based pharmaceutical tablet manufacturing: a cohesive analysis at three different levels. Green chemistry. 2013;15(11):3039-48.

6. Verlinden A, Boone L, De Soete W, Dewulf J. Environmental impacts of drug products: The effect of the selection of production sites in the supply chain. Sustainable Production and Consumption. 2024;52:1-11.

7. Aus der Beek T, Weber FA, Bergmann A, Hickmann S, Ebert I, Hein A, Küster A. Pharmaceuticals in the environment—Global occurrences and perspectives. Environmental toxicology and chemistry. 2016;35(4):823-35.

8. Wilkinson JL, Boxall ABA, Kolpin DW, Leung KMY, Lai RWS, Galbán-Malagón C, et al. Pharmaceutical pollution of the world’s rivers. Proceedings of the National Academy of Sciences. 2022;119(8):e2113947119.

9. Raju G, Sarkar P, Singla E, Singh H, Sharma R. Comparison of Environmental Sustainability of Pharmaceutical Packaging. Perspectives in Science. 2016;8.

10. Page RL, 2nd, Linnebur SA, Bryant LL, Ruscin JM. Inappropriate prescribing in the hospitalized elderly patient: defining the problem, evaluation tools, and possible solutions. Clin Interv Aging. 2010;5:75-87.

11. Mahase E. Overprescribing: 10% of items dispensed in primary care are inappropriate, review finds. Bmj. 2021;374:n2338.

12. Daughton CG, Ruhoy IS. Lower-dose prescribing: Minimizing “side effects” of pharmaceuticals on society and the environment. Science of The Total Environment. 2013;443:324-37.

13. Eii MN, Walpole S, Aldridge C. Sustainable practice: Prescribing oral over intravenous medications. Bmj. 2023;383:e075297.

14. Janson C, Henderson R, Löfdahl M, Hedberg M, Sharma R, Wilkinson AJK. Carbon footprint impact of the choice of inhalers for asthma and COPD. Thorax. 2020;75(1):82-4.

15. Kirchherr J, Reike D, Hekkert MP. Conceptualizing the Circular Economy: An Analysis of 114 Definitions. SSRN Electronic Journal. 2017;127.

16. Gnjidic D, Johansson M, Meng DM, Farrell B, Langford A, Reeve E. Achieving sustainable healthcare through deprescribing. Cochrane Database Syst Rev. 2022;10(10):ED000159.

17. Manyara AM, Purvis A, Ciani O, Collins GS, Taylor RS. Sample size in multistakeholder Delphi surveys: at what minimum sample size do replicability of results stabilize? Journal of Clinical Epidemiology. 2024;174.

18. Kilroy D, Driscoll P. Determination of required anatomical knowledge for clinical practice in emergency medicine: national curriculum planning using a modified Delphi technique. Emerg Med J. 2006;23(9):693-6.

19. Fitch K, Bernstein SJ, Aguilar MD, Burnand B, LaCalle JR, Lázaro P, et al. The RAND/UCLA Appropriateness Method User’s Manual. Santa Monica: RAND; 2001.

20. Patient Federation Netherlands, the Dutch College of General Practitioners (NHG), the National Association of General Practitioners (LHV), the Federation of Medical Specialists (FMS), the Royal Society for Advancement of Pharmacy (KNMP), Healthcare insurances Netherlands (ZN). Leidraad Verantwoord Wisselen Medicijnen 2024.

21. Stichting Werkgroep Antibiotica Beleid (SWAB). SWAB Guidelines 2025 [Available from: <https://swab.nl/nl/richtlijnen-swab>.

22. World Health Organization. ATC - DDD Toolkit 2025 [Available from: <https://www.who.int/tools/atc-ddd-toolkit/atc-classification>.

## **Supplement 2: DELPHISTAR Reporting guidelines for Delphi-studies**


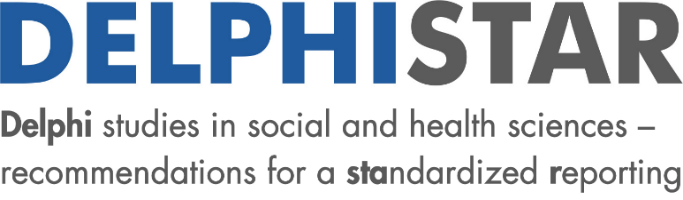


**Delphi studies in social and health sciences – recommendations for an interdisciplinary standardized reporting (DELPHISTAR).**

From: Niederberger, M., Schifano, J., Deckert, S., Hirt, J., Homberg, A., Köberich, S., Kuhn, R., Rommel, A., Sonnberger, M. & the DEWISS network (2024). Delphi studies in social and health sciences—Recommendations for an interdisciplinary standardized reporting (DELPHISTAR). Results of a Delphi-study. *PLoS ONE 19(8):* e0304651. <https://doi.org/10.1371/journal.pone.0304651>

| Topic | Section | Item | Checklist Item | Location | Exemplary wording |
| --- | --- | --- | --- | --- | --- |
| I  Title and Abstract |  | 1 | Identification as a Delphi-study in the title | Title | What is a public health intervention? Results of a Delphi-study. |
|  |  | 2 | Identification as a Delphi-study in the abstract | Abstract | A Delphi-study was selected to answer the research question. |
|  |  | 3 | Structured abstract | Abstract | e.g., background, method, results and discussion |
| II  Context | **Formal** | 4 | Information about the sources of funding | Funding | The Delphi-study was funded by [SOURCE]. |
|  |  | 5 | Information about the team of authors and/or researchers (e.g., discipline, institution) | Identifying relevant (de)prescribing interventions | The Delphi-study was conducted by an interdisciplinary team with representatives from medicine, public health, and health promotion. |
|  |  | 6 | Information about method consulting | Design | The study group was advised by experts from [INSTITUTION] regarding statistics.  Or:  No consulting in regard to method took place. |
|  |  | 7 | Information about the project background | Introduction | The Delphi-study was part of a mixed-methods study on [AIM]. |
|  |  | 8 | Information about the study protocol | Suppl 1 | The study protocol is available at [LINK]. |
|  | **Content** | 9 | Justification of the chosen method (Delphi) to answer the research question | Introduction | The Delphi method is appropriate for answering the research question because it systematically gathers the judgments of different expert groups and can identity agreement and disagreement. |
|  |  | 10 | Aim of the Delphi-study (e.g., consensus, forecasting) | Introduction | The aim of the Delphi-study is to find consensus on criteria to define a public health intervention. |
| III  Method | **Body & Integration of knowledge** | 11 | Identification and elucidation of relevant expertise, spheres of experience, and perspectives (e.g., theory, practice, affected groups, disciplines) | Study population | The experts represent the sciences and clinical practice because [REASON]. |
|  |  | 12 | Handling of knowledge, expertise and perspectives which are missing or have been deliberately not integrated | NA | If it is not possible to recruit experts specialized in [AREA], this will be openly communicated to the other experts during the Delphi-study. |
|  |  | 13 | Basic definition of expert^1^ | Study population | A person who has been active in the area for at least [NUMBER] years is considered to be an expert. |
|  | **Delphi variant and modifications** | 14 | Identification of the type of Delphi variant and potential modifications (e.g., classic Delphi, real-time Delphi, group Delphi) | Design | A classic Delphi-study was used [LITERATURE REFERENCE]. |
|  |  | 15 | Justification of the Delphi variant and modifications, including during the Delphi-study, if applicable | Methods (data collection/ analysis) | If the willingness to participate clearly decreases between the first and second round, a third round will not be held. |
|  | **Sample of experts** | 16 | Selection criteria for the experts (per round, per expert group if applicable) | Study population | All of the experts who met the definition were invited to the first round.  All of the experts who completed the previous round were invited to participate in the subsequent round. |
|  |  | 17 | Identification of the experts | Study population | The experts were identified based on publications in [DATABASE]. |
|  |  | 18 | Information about recruiting and any subsequent recruiting of experts | Delphi-study | The experts were informed about the Delphi-study and invited to participate. |
|  | **Survey** | 19 | Elucidation of the content development for the questionnaire^2^ | Delphi-study | The questionnaire was developed based on the results of systematic reviews [LITERATURE REFERENCE]. |
|  |  | 20 | Description of the questionnaire (content and structure) | Delphi-study Suppl 4 | The questionnaire was divided into three segments on [TOPICS]. The statements made in the questionnaire were evaluated using standardized items, with the option to comment in free-text boxes. |
|  | **Delphi-rounds** | 21 | Number of Delphi-rounds | Delphi-study | Three Delphi-rounds were held. |
|  |  | 22 | Information about the aims of the individual Delphi-rounds | Appropriateness of (de-) prescribing interventions & Prioritizing (de)prescribing interventions | The first Delphi-round focused on exploring relevant aspects. These aspects were then presented to the experts in the second Delphi-round for standardized evaluation. |
|  |  | 23 | Disclosure and justification of the criterion for discontinuation | Delphi-study | The number of rounds was defined in advance to be a maximum of three rounds. |
|  | **Feedback** | 24 | Information about what data was reported back per round | Appropriateness of (de-) prescribing interventions & Prioritizing (de)prescribing interventions | In terms of feedback, we shared the statistical results plus the summary of the open responses. |
|  |  | 25 | Information on how the results of the previous Delphi-round were fed back to the experts surveyed (e.g., via frequencies, mean values, measures of dispersion, listing of comments) | Appropriateness of (de-) prescribing interventions & Prioritizing (de)prescribing interventions | Mean values, standard deviations and percentage frequency distributions were reported. |
|  |  | 26 | Information on whether feedback was differentiated by specific groups (e.g., by field of expertise, institutional affiliation) | Appropriateness of (de-) prescribing interventions & Prioritizing (de)prescribing interventions | The feedback was aggregated across all expert groups. |
|  |  | 27 | Information about how dissent and unclear results were handled | Appropriateness of (de-) prescribing interventions & Prioritizing (de)prescribing interventions | The results showing dissent were presented again for evaluation in the next Delphi-round. |
|  | **Data analysis** | 28 | Disclosure of the quantitative and qualitative analytical strategy | Appropriateness of (de-) prescribing interventions & Prioritizing (de)prescribing interventions | The quantitative items were descriptively analyzed. The open-ended items were analyzed using thematic analysis [LITERATURE REFERENCE]. |
|  |  | 29 | Definition and measurement of consensus | Appropriateness of (de-) prescribing interventions & Prioritizing (de)prescribing interventions | Consensus was defined as percentage agreement, meaning that agreement was assumed if at least 80% of the respondents agreed on an item. |
|  |  | 30 | Information on group-specific analysis or weighting of experts (e.g., theory vs. practice, discipline-specific analysis) | NA | In the analysis, the mean values for percent agreement are weighted for each expert group in terms of the number of group members. |
| IV  Results | **Delphi process** | 31 | Illustration of the Delphi-study (e.g., in a flow chart) | Fig 1  Fig 3 | A summary of the Delphi-study is illustrated in a flow chart (Figure 1). |
|  |  | 32 | Information about special aspects during the Delphi-study (e.g., deviations from the intended approach with justification) | NA | During the Delphi-study the political discussion mentioned climate change and the effects on health. It is possible that this influenced the experts' responses. |
|  |  | 33 | Number of experts per round (both invited and participating) | Fig 1  Delphi-panel | The number of experts participating in the first Delphi-round was [NUMBER], and the number of experts in the second round was [NUMBER]. This corresponds to a response rate of [NUMBER]% in the first round and [NUMBER]% in the second round. |
|  | **Results** | 34 | Presentation of the results for each Delphi-round and the final results | Fig 3  Table 2 & 3 Evaluating appropriateness of (de)prescribing interventions & Prioritizing (de)prescribing interventions | In the first Delphi-round [NUMBER]% of the experts agreed, in the second [NUMBER]%, and in the third [NUMBER]%. |
| V Discussion | **Quality of findings** | 35 | Highlighting the findings from the Delphi-study | Discussion | The central findings can be summarized as follows: [STATE FINDINGS]. |
|  |  | 36 | Validity of the results (e.g., transferability of the findings) | Discussion | The results are not transferable to other countries due to different legal regulations. |
|  |  | 37 | Reliability of the results (e.g., split half, inter-rater reliability) | Discussion | The responses in the free-text comments were analyzed by two independent reviewers [SPECIFY]. |
|  |  | 38 | Reflection on potential limitations (e.g., number of experts, response bias) | Discussion | The results are to be viewed critically with regard to the composition of the panel because [REASONS]. |

^1^ “Experts” are the participants; these can be people from academia, practice, or representatives of lived experience (e.g., patients, family members).

^2^ The term “questionnaire” stands for the survey instrument regardless of whether quantitative or qualitative items are integrated or weighted.

## **Supplement 3: Literature Search**

**Medline**

(* Sustainable Development/ OR * Conservation of Natural Resources/ OR * Carbon Footprint/ OR * Waste Management/ OR * Recycling/ OR * Climate Change/ OR * Greenhouse Gases/ OR (((environmental* OR climate*) ADJ3 (sustainab* OR impact* OR protect* OR emergenc* OR conscious*)) OR environmental*-friend* OR ((green OR sustainab* OR greening) ADJ3 (medication* OR medicines* OR medication* OR pharmac*)) OR ((eco-direct* OR ecodirect* OR sustainab* OR low*-dos* ) ADJ3 prescri*) OR ((carbon*) ADJ3 (footprint* OR neutal*)) OR decarboni* OR (climate* ADJ3 change*) OR green-house-gas* OR greenhouse-gas* OR ((reduc* OR decreas* OR minimi* OR management* OR managing OR combat* OR prevent*) ADJ3 (waste OR wastage* OR pollution* OR ecotoxic* OR eco*-toxic* OR environment*-harm*)) OR recycl* OR sustainab* OR green*-prescri* OR greener OR eco*-friend*).ti.) AND (* Drug Therapy/ OR * Biopharmaceutics/ OR * Drug Compounding/ OR * Dosage Forms/ OR * Drug Packaging/ OR * Inappropriate Prescribing/ OR * Prescription Drug Misuse/ OR * Drug Misuse/ OR * Prescription Drug Overuse/ OR * Prescriptions/ OR * Prescription Drugs/ OR * Pharmacy/ OR * Pharmacists/ OR ((drug ADJ3 (therap* OR formulat* OR dosage* OR form* OR package*)) OR medication* OR medicines* OR medicinal* OR pharmaceutic* OR pharmacy OR pharmacies OR pharmacist* OR pharmacotherap* OR prescri* OR deprescri* OR overprescri* OR (swallow ADJ3 pill*) OR ((solid OR liquid) ADJ3 (drug* OR formulation*))).ti.) NOT (drug industry/ OR (industr* OR company OR companies).ti.) NOT (news OR congres* OR abstract* OR book* OR chapter* OR dissertation abstract*).pt. AND (english.la. OR dutch.la.) AND 2005:2025.(sa_year).

limit 1 to abstracts

**embase**

('environmental sustainability'/mj OR 'sustainable development'/mj OR 'environmental impact'/mj OR 'environmental protection'/mj OR 'carbon footprint'/mj OR 'waste management'/mj OR recycling/mj OR 'waste minimization'/mj OR 'carbon neutrality'/mj OR 'climate change'/mj OR ' greenhouse gas'/mj OR 'greenhouse gas emission'/mj OR (((environmental* OR climate*) NEAR/3 (sustainab* OR impact* OR protect* OR emergenc* OR conscious*)) OR environmental*-friend* OR ((green OR sustainab* OR greening) NEAR/3 (medication* OR medicines* OR medication* OR pharmac*)) OR ((eco-direct* OR ecodirect* OR sustainab* OR low*-dos* ) NEAR/3 prescri*) OR ((carbon*) NEAR/3 (footprint* OR neutal*)) OR decarboni* OR (climate* NEAR/3 change*) OR green-house-gas* OR greenhouse-gas* OR ((reduc* OR decreas* OR minimi* OR management* OR managing OR combat* OR prevent*) NEAR/3 (waste OR wastage* OR pollution* OR ecotoxic* OR eco*-toxic* OR environment*-harm*)) OR recycl* OR sustainab* OR green*-prescri* OR greener OR eco*-friend*):ti) AND ('drug therapy'/mj OR pharmaceutics/mj OR 'drug formulation'/mj OR 'drug dosage form'/mj OR 'drug packaging'/mj OR 'prescribing practice'/mj OR 'prescription drug misuse'/exp/mj OR 'unnecessary prescribing'/mj OR 'medication overuse'/mj OR 'prescription'/mj OR 'pharmacy practice'/mj OR pharmacist/mj OR ((drug NEAR/3 (therap* OR formulat* OR dosage* OR form* OR package*)) OR medication* OR medicines* OR medicinal* OR pharmaceutic* OR pharmacy OR pharmacies OR pharmacist* OR pharmacotherap* OR prescri* OR deprescri* OR overprescri* OR (swallow NEAR/3 pill*) OR ((solid OR liquid) NEAR/3 (drug* OR formulation*))):ti) NOT ('drug industry'/mj OR (industr* OR company OR companies):ti) NOT [conference abstract]/lim AND [abstracts]/lim AND ([english]/lim OR [dutch]/lim) AND [2005-2025]/py

**Web of science**

(TI=(((environmental* OR climate*) NEAR/2 (sustainab* OR impact* OR protect* OR emergenc* OR conscious*)) OR environmental*-friend* OR ((green OR sustainab* OR greening) NEAR/2 (medication* OR medicines* OR medication* OR pharmac*)) OR ((eco-direct* OR ecodirect* OR sustainab* OR low*-dos* ) NEAR/2 prescri*) OR ((carbon*) NEAR/2 (footprint* OR neutal*)) OR decarboni* OR (climate* NEAR/2 change*) OR green-house-gas* OR greenhouse-gas* OR ((reduc* OR decreas* OR minimi* OR management* OR managing OR combat* OR prevent*) NEAR/2 (waste OR wastage* OR pollution* OR ecotoxic* OR eco*-toxic* OR environment*-harm*)) OR recycl* OR sustainab* OR green*-prescri* OR greener OR eco*-friend*)) AND (TI=((drug NEAR/2 (therap* OR formulat* OR dosage* OR form* OR package*)) OR medication* OR medicines* OR medicinal* OR pharmaceutic* OR pharmacy OR pharmacies OR pharmacist* OR pharmacotherap* OR deprescri* OR overprescri* OR (swallow NEAR/2 pill*) OR ((solid OR liquid) NEAR/2 (drug* OR formulation*)))) AND (AB=(drug OR medic* OR pharm*)) NOT TI=((industr* OR company OR companies)) NOT DT=(Meeting Abstract OR Meeting Summary) AND LA=(English OR dutch) AND PY=(2005-2025)

## **Supplement 4: Questionnaire**

This questionnaire was conducted in Dutch but has been translated to English for publication.

### Round 1/2

**Introduction**

Dear healthcare professional,

This questionnaire presents sustainability interventions for medications. We ask you to evaluate these interventions for their appropriateness for implementation in Dutch hospitals in order to reduce the environmental impact of healthcare.

*Background*

The most commonly used medications (or medication classes) have been selected for both clinical and outpatient care from Dutch hospitals. These medications are linked to potential sustainability interventions based on literature, expert opinion and existing (de)prescribing guidelines, focusing on options for:

- Deprescribing: reducing prescriptions and/or prescribing lower dosages of medications.
- Switching to more sustainable administration forms: Such as oral versus parenteral routes, and the choice of inhaler types.

*Research Objectives*

In this study, you, a panel of physicians and pharmacists, will decide which sustainability interventions are appropriate for implementation in Dutch hospitals for each medication (class). The highest ranked interventions will included in the Citrienfonds program “Together Greening Healthcare”, meaning that Dutch hospitals will implement these interventions over the next two years to reduce the environmental impact of healthcare.

*Structure of the Study*

The interventions will be evaluated in multiple rounds. In this round, we ask you to assess all possible strategies. [Strategies where no consensus has been reached will be revisited in a subsequent round. You will see the group results and we ask you to adjust your opinion based on this feedback. This process will be repeated up to two times (a maximum of three questionnaires) to reach consensus on appropriate sustainability interventions for medications prescribed in Dutch hospitals].

*Practical Information*

- Participation is entirely voluntary. You can withdraw from the study at any time without providing a reason. Your responses will still be used in the study.
- We will only use your contact information for distributing the questionnaire and communicating with you. These details will be deleted after the study.
- Your responses are anonymous and cannot be linked to your identity. Your responses will also not be visible to other participants. We will store your responses for a period of 10 years in a secure environment at Erasmus MC.
- You can save your progress and return to the questionnaire at any time.
- You will have two weeks to complete each round of the questionnaire.
- We ask you to complete the questionnaires. For every participant who completes the questionnaire, [one tree/two trees] will be planted.

By proceeding with the questionnaire, you agree to the above information. If you have any questions, please contact us.

Thank you for your participation!

**Background information:**

Since the questionnaire is anonymous, we kindly ask you to briefly answer a few questions about your background:

What is your role? [multiple answers possible]

- Medical specialist
- Resident
- Hospital pharmacist
- Outpatient pharmacist
- Pharmacy resident
- Other: [open text]

*Within which specialty are you working?

- Anesthesiology
- Cardiology
- Chirurgy
- Dermatology
- Emergency medicine
- Gastroenterology
- Geriatrics
- Gynecology
- Internal Medicine
- Intensive Care
- Neurology
- Ophthalmology
- Orthopedics
- Otorhinolaryngology
- Pediatrics
- Pulmonology
- Rheumatology
- Urology
- Other: [open text]

**Type of hospital:**

- Academic
- Teaching hospital
- General
- Other: [open text]

**Sustainability interventions for medications** [only in round 1]:

Several strategies can be used to make medication use more sustainable in Dutch hospitals. This program focuses on two types of sustainability interventions:

1. Deprescribing: Reducing unnecessary medication use by not starting, lowering the dosage, tapering, or stopping medications.
2. Switching to more sustainable administration forms: Choosing the most sustainable variant of a medication, related to the route of administration (e.g., oral versus intravenous) and form (e.g., tablets versus liquids, or different types of inhalers).

*Selection of Medications:*

In the questionnaire, you will be asked to evaluate sustainability interventions for the 20 most prescribed medications (classes) in in- and outpatient care from Dutch hospitals. A pre-selection has been made based on the following criteria:

- Contains an active pharmaceutical ingredient.
- Regularly prescribed in Dutch hospitals.
- Modifiable based on the KNMP guideline ‘Responsible Switching’.

**Outpatient care – deprescribing**

The interventions presented to you on this page concern the ‘deprescribing’ of medications used in the home setting*.

For each medication group, you are asked to assess the suitability of a sustainability initiative for implementation in Dutch hospitals. When evaluating, please consider:

- The effectiveness and safety of the treatment remain guaranteed;
- The environmental impact of the treatment is reduced, for example by reduced dosage and/or less packaging material;
- The initiative can be implemented in Dutch hospitals.

Other factors you may consider during your evaluation include:

- The level of scientific evidence or practical experience demonstrating that the initiative is appropriate for reducing environmental impact;
- How well the interventions fit into existing care pathways and/or their ease of implementation.

“This [intervention] is appropriate for implementation in Dutch hospitals to reduce environmental impact”.

1. Strongly disagree
2. Disagree
3. Slightly Disagree
4. Neither Agree nor Disagree
5. Slightly Agree
6. Agree
7. Strongly Agree

Do you have any questions or comments regarding the abovementioned interventions?

[open text]

Besides the interventions mentioned above, are you aware of any other opportunities for deprescribing in outpatient care to reduce the environmental impact of Dutch hospitals?

[open text]

* Deprescribing refers to reducing unnecessary medication use. This includes not starting medication, reducing dosage, tapering off, and/or discontinuing medication.

**Outpatient care – switching to the most sustainable administration form**

The interventions presented to you on this page concern the ‘switch to the most sustainable dosage forms’ of medications used in the home setting*.

*As “outpatient care – deprescribing”*

* 'Switch to the most sustainable dosage form' means choosing the most sustainable variant of a medication in terms of route of administration (e.g., oral versus intravenous) and dosage form (e.g., tablets versus liquids, or different types of inhalers).

***Inpatient care – deprescribing***

The interventions presented to you on this page concern the ‘deprescribing’ of medications used during hospitalization*.

*As “outpatient care – deprescribing”*

* Deprescribing refers to reducing unnecessary medication use. This includes not starting medication, reducing dosage, tapering off, and/or discontinuing medication.

**Inpatient care – switching to the most sustainable administration form**

The interventions presented to you on this page concern the ‘switch to the most sustainable dosage forms’ of medications used during hospitalization*.

*As “outpatient care – deprescribing”*

* 'Switch to the most sustainable dosage form' means choosing the most sustainable variant of a medication in terms of route of administration (e.g., oral versus intravenous) and dosage form (e.g., tablets versus liquids, or different types of inhalers).

**Final remarks**

Do you have any comments or suggestions regarding this questionnaire that we should consider for the next round?

[Text field]

Thank you for completing this questionnaire; your input will help select sustainability interventions for medications! As a thank you, we will plant a tree/two trees for your participation.

You will receive feedback on the results of this round and an invitation to participate in the next round via email.

### Round 3

Dear healthcare professional,

You have reached the third and final round of the Delphi study on sustainability interventions for medications in Dutch hospitals.

In this final round, we ask you to prioritize the interventions that were deemed appropriate in previous rounds. Which of these, in your opinion, deserve the highest priority for implementation and scaling up in the Netherlands? Please consider criteria such as environmental impact, feasibility, scalability, and urgency.

The highest-scoring interventions will be implemented in Dutch hospitals within the "Together Greening Healthcare" program, with the goal of reducing the environmental impact of healthcare.

So far, 51 healthcare professionals have fully participated in round 1, and 49 in round 2. As a token of appreciation, we will plant at least 149 trees. In this final round, we will plant 3 additional trees for each fully completed questionnaire!

Thank you in advance for your contribution to this important step toward more sustainable healthcare.

*Practical Information*

- Participation in the study is entirely voluntary, and your responses will be processed anonymously.
- We will store your answers for a period of 10 years in a secure environment at Erasmus MC.
- You can save your progress and return to the questionnaire at any time.
- You have until July 20 to complete the questionnaire.
- Thank you for your dedication!

**Background information:**

Since the questionnaire is anonymous, we kindly ask you to briefly answer a few questions about your background:

What is your role? [multiple answers possible]

- Medical specialist
- Resident
- Hospital pharmacist
- Outpatient pharmacist
- Pharmacy resident
- Other: [open text]

*Within which specialty are you working?

- Anesthesiology
- Cardiology
- Chirurgy
- Dermatology
- Emergency medicine
- Gastroenterology
- Geriatrics
- Gynecology
- Internal Medicine
- Intensive Care
- Neurology
- Ophthalmology
- Orthopedics
- Otorhinolaryngology
- Pediatrics
- Pulmonology
- Rheumatology
- Urology
- Other: [open text]

**Type of hospital:**

- Academic
- Teaching hospital
- General
- Other: [open text]

**Outpatient care – deprescribing**

The interventions presented to you on this page concern the ‘deprescribing’ of medications used in the home setting.

In the previous rounds, we have presented you with fifteen interventions related to the ‘deprescribing’ of medications in outpatient care. There was consensus among the participants on ten of these interventions: they were deemed appropriate for implementation in Dutch hospitals.

In this final round, we ask you to prioritize these ten interventions. Which interventions, in your opinion, deserve the highest priority for implementation and scaling up in Dutch hospitals to reduce the environmental impact of healthcare? Please consider criteria such as environmental impact, feasibility, scalability, and urgency.

*Which of the following interventions is most appropriate for implementation in Dutch hospital care in order to reduce environmental impact? Select the top 5:*

* Deprescribing refers to reducing unnecessary medication use. This includes not starting medication, reducing dosage, tapering off, and/or discontinuing medication.

**Outpatient care – switching to the most sustainable administration form**

The interventions presented to you on this page concern the ‘switch to the most sustainable dosage forms’ of medications used in the home setting*.

*As “outpatient care – deprescribing”*

* 'Switch to the most sustainable dosage form' means choosing the most sustainable variant of a medication in terms of route of administration (e.g., oral versus intravenous) and dosage form (e.g., tablets versus liquids, or different types of inhalers).

**Inpatient care – deprescribing**

The interventions presented to you on this page concern the ‘deprescribing’ of medications used during hospitalization*.

*As “outpatient care – deprescribing”*

* Deprescribing refers to reducing unnecessary medication use. This includes not starting medication, reducing dosage, tapering off, and/or discontinuing medication.

**Inpatient care – switching to the most sustainable administration form**

The interventions presented to you on this page concern the ‘switch to the most sustainable dosage forms’ of medications used during hospitalization*.

*As “outpatient care – deprescribing”*

* 'Switch to the most sustainable dosage form' means choosing the most sustainable variant of a medication in terms of route of administration (e.g., oral versus intravenous) and dosage form (e.g., tablets versus liquids, or different types of inhalers).

**Final remark**

Thank you for completing this questionnaire; your input will help to select and prioritize sustainability interventions for medications, which will be implemented during the implementation program! We will plant three tree for your participation in this survey.

## **Supplement 5: Selected medication classes for prescribing interventions**

**Table 1: Overview of selected medication classes for (de)prescribing interventions, based on the most frequently prescribed drugs in Dutch inpatient and outpatient care.**

| ATC Level 2 | Therapeutic group | ATC Level 4 | Chemical subgroup | Outpatient Top-20 | Inpatient Top-20^*1^ |
| --- | --- | --- | --- | --- | --- |
| A02 | Drugs for acid related disorders | A02BC | Proton pump inhibitors | X | X |
| A03 | Drugs for functional GI disorders | A03FA | Propulsives | X | X |
| A04 | Antiemetics and antinauseants | A04AA | 5-HT3 antagonists |  | X |
| A10 | Drugs used in diabetes | A10BA | Biguanides |  | X |
|  |  | A10BB | Sulfonylureas |  | X |
| B01 | Antithrombotic agents | B01AB | Heparins |  | X |
|  |  | B01AC | Platelet aggregation inhibitors | X | X |
|  |  | B01AF | Direct factor Xa inhibitors | X | X |
|  |  | B01AX | Other antithrombotic agents |  | X |
| B03 | Antianaemic preparations | B03AA | Iron bivalent, oral | X | X |
|  |  | B03XA | Erythropoietic growth factors |  | X |
| C03 | Diuretics | C03AA | Thiazides, plain | X |  |
|  |  | C03CA | Sulfonamides, plain |  | X |
|  |  | C03DA | Aldosterone antagonists |  | X |
| C07 | Beta blocking agents | C07AB | Beta-blockers, selective | X | X |
| C08 | Calcium channel blockers | C08CA | Dihydropyridine derivatives | X | X |
| C09 | Agents acting on renin-angiotensin system | C09AA | ACE inhibitors, plain | X |  |
|  |  | C09CA | Angiotensin II receptor blockers, plain | X |  |
| C10 | Lipid modifying agents | C10AA | HMG CoA reductase inhibitors | X | X |
| G04 | Urologicals | G04CA | Alpha-adrenoreceptor antagonists | X |  |
| H02 | Corticosteroids for systemic use | H02AB | Glucocorticoids | X |  |
| J01 | Antibacterials for systemic use | J01AA | Tetracyclines | X | X |
|  |  | J01D_ | Cephalosporins |  | X |
|  |  | J01C_ | Penicillins | X | X |
| M01 | Anti-inflammatory products | M01A | NSAIDs | X | X |
| N02 | Analgesics | N02A | Opioids | X | X |
|  |  | N02BE | Anilides | X | X |
| N05 | Psycholeptics | N05BA | Benzodiazepines |  | X |
| R03 | Drugs for obstructive airway diseases | R03BA | Glucocorticoids | X |  |
|  |  | R03AC | Beta-2-adrenoreceptor agonists | X |  |
|  |  | R03AK | Beta-2 agonists with corticosteroids |  | X |

*^1^ Based on top-20 administrations of four hospitals that did not fully overlap, so >20 classes are shown.

## **Supplement 6: Appropriateness and prioritization of (de)prescribing interventions**

**Table 1: Interventions with consensus on appropriateness in Delphi-round 1 (n=30)**

| Therapeutic group | | Medication class | Setting | Type | Prescribing intervention | Appropriateness Median (DI) |
| --- | --- | --- | --- | --- | --- | --- |
| A02 | Drugs for acid related disorders | Proton pump inhibitors (PPIs) | Inpatient | D | Avoid routine PPI initiation in low-risk patients | 6 (0.2) |
|  |  |  | Inpatient | D | Stop chronically used PPIs without indication | 6 (0.2) |
|  |  |  | Outpatient |  |  | 7 (0.2) |
|  |  |  | Inpatient | S | Prescribe oral pantoprazole instead of intravenous | 6 (0.2) |
| A04 | Antiemetics and antinauseants | 5-HT3 antagonists | Inpatient | S | Prescribe oral ondansetron or granisetron instead of intravenous | 6 (0.3) |
| B01 | Antithrombotic agents | Antithrombotic agents | Inpatient | D | Actively monitor stop dates of combination therapy during admission | 6 (0.0) |
|  |  |  | Outpatient | D | Monitor stop dates of combination therapy and share indication/duration with primary care providers | 6 (0.2) |
|  |  |  |  | D | Short-term DAPT after drug-eluting stenting in patients with ACS. | 6 (0.2) |
| B03 | Antianemic preparations | Erythropoietic growth factors | Outpatient | S | Administer epoetin beta weekly instead of 3–7 times/week in cancer patients | 6 (0.1) |
| C03 | Diuretics | Loop diuretics | Inpatient | S | Administer loop diuretics as bolus injection instead of infusion | 6 (0.5) |
|  |  |  |  | S | Prescribe oral loop diuretics instead of intravenous in non-acute situations | 6 (0.3) |
| C10 | Lipid modifying agents | HMG CoA reductase inhibitors | Inpatient | D | Discontinue statins in advanced disease | 7 (0.2) |
|  |  |  | Outpatient | D | Taper statins in patients with low CV risk | 6 (0.3) |
| G04 | Urologicals | Alpha- receptor antagonists | Outpatient | D | Stop alpha-1 blockers after 6 months in men with LUTS | 6 (0.3) |
| J01 | Antibacterials for systemic use | Various | Inpatient | D | Add a stop date when initiating antibiotic treatment (course) | 6 (0.2) |
|  |  |  |  | D | Add a stop date when initiating prophylactic antibiotics | 6 (0.5) |
|  |  |  |  | S | Administer antibiotics as bolus injection instead of infusion | 6 (0.2) |
|  |  |  |  | S | Initiate oral antibiotics with good bioavailability | 6 (0.2) |
| M01 | Anti-inflammatory drugs | NSAIDs | Inpatient | D | Replace NSAIDs with adequate paracetamol dosing | 6 (0.3) |
| N02 | Analgesics | Opioids | Inpatient | S | Prescribe oral morphine instead of intravenous morphine | 6 (0.2) |
|  |  |  |  | S | Prescribe oral morphine instead of oxycodone | 6 (0.3) |
|  |  |  |  | D | Reduce opioid prescriptions at discharge by standardized pain plans | 6 (0.2) |
|  |  |  |  | D | Reduce opioid prescriptions at discharge by including a stop date | 6 (0.2) |
|  |  |  | Outpatient | D | Reduce opioid use by communicating intended treatment duration with primary providers | 6 (0.2) |
|  |  |  | Inpatient | D | Stepwise pain management (no routine opioid prescribing) after caesarean sec. | 6 (0.3) |
| N05 | Psycholeptics | Benzodiazepines | Inpatient | D | Discontinuation of benzodiazepines in geriatric inpatients | 6 (0.3) |
|  |  |  |  | S | Prescribe oral lorazepam instead of intravenous as premedication | 6 (0.0) |
| R03 | Drugs for obstructive airway diseases | Various | Outpatient | S | Initiate dry powder inhalers instead of metered-dose inhalers where possible | 6 (0.1) |
|  |  | Various | Outpatient | S | Switch from metered-dose inhalers to dry powder inhalers instead | 6 (0.1) |
|  |  | Various | Outpatient | D | Re-evaluate inhaled corticosteroids after 1 year in COPD | 6 (0.3) |

D: deprescribing intervention; S: switch to most sustainable dosage form; DI: disagreement index.

ACS: Acute Coronary Syndrome, COPD: Chronic Obstructive Pulmonary Disease, CV: Cardiovascular, DAPT: double anti-platelet therapy, LUTS: Lower urinary tract symptoms, MI: myocardial infarction, NSAIDs: nonsteroidal anti-inflammatory drugs, PPIs: proton pump inhibitors.

**Table 2: Interventions re-assessed in Delphi-round 2 due to no consensus in round 1 (n=8)**

| Therapeutic group | | Medication class | Setting | Type | Prescribing intervention | Appropriateness Median (DI) | |
| --- | --- | --- | --- | --- | --- | --- | --- |
| A10 | Drugs used in diabetes | Blood glucose lowering drugs | Outpatient | D | Adjust therapy (e.g. stop combination or reduce dose) if hypoglycemia risk or targets achieved | 5 (0.3) | 5 (0.7) |
|  |  |  | Inpatient | D | Reconcile/taper dosing based on HbA1c and glucose | 5 (0.4) | 4 (0.7) |
| C02 | Anti-hypertensives | Various | Outpatient | D | Taper preventive antihypertensives in patients with low CV risk | 5 (0.3) | 5 (0.7) |
| C03 | Diuretics | Thiazides | Outpatient | D | Review chronic diuretic use to taper/stop if clinically appropriate | 5 (0.7) | 5 (0.7) |
| C07 | Beta-blocking agents | Beta-blocking agents | Outpatient | D | Stop beta-blockers post-MI in patients with normal cardiac function (LVEF >50%) | 5 (0.7) | 6 (0.5) |
|  |  |  | Inpatient | D | Do not initiate beta-blockers post-MI in patients with normal cardiac function | 5 (0.4) | 6 (0.3) |
| M01 | Anti-inflammatory drugs | NSAIDs | Outpatient | D | Replace NSAIDs with adequate paracetamol dosing | 5 (0.3) | 6 (0.5) |
| N05 | Psycholeptics | Benzodiazepines | Inpatient | D | Avoid initiating benzodiazepines; use non-pharmacological interventions | 5 (0.3) | 5 (0.3) |

D: deprescribing intervention; S: switch to most sustainable dosage form; DI: disagreement index.

CV: Cardiovascular, LVEF: Left Ventricular Ejection Fraction, MI: myocardial infarction, NSAIDs: nonsteroidal anti-inflammatory drugs.

**Table 3: Interventions with consensus on appropriateness in Delphi-round 1 but re-assessed in round 2 due to adjustments based on participants’ comments (n=5)**

| Therapeutic group | | Medication class | Setting | Type | Prescribing intervention | Appropriateness Median (DI) | |
| --- | --- | --- | --- | --- | --- | --- | --- |
| A03 | Drugs for functional disorders | Propulsives | Inpatient | S | Prescribe *enteral* metoclopramide instead of intravenous | 6 (0.3) | 6 (0.2) |
| B01 | Antithrombotic agents | Antithrombotic agents | Outpatient | D | Stop antiplatelets for primary prevention *in patients >70 years* | 6 (0.3) | 5 (0.3) |
| B03 | Antianemic preparations | Iron preparations | Outpatient | S | Administer oral iron tablets instead of liquid iron, *preferably every other day* | 6 (0.0) | 6 (0.1) |
|  |  |  | Inpatient |  |  | 6 (0.3) | 6 (0.2) |
| N02 | Analgesics | Other analgesics and antipyretics | Inpatient | S | Prescribe oral paracetamol instead of intravenous *(paracetamol challenge)* | 6 (0.3) | 6 (0.2) |

*Adjustments made after the first Delphi-round in italics.*

D: deprescribing intervention; S: switch to most sustainable dosage form; DI: disagreement index.

**Table 4: Interventions added in Delphi-round 2 based on participants’ comments (n=8)**

| Therapeutic group | | Medication class | | Setting | | Type | | Prescribing intervention | | Appropriateness Median (DI) | | |
| --- | --- | --- | --- | --- | --- | --- | --- | --- | --- | --- | --- | --- |
| A06 | Drugs for constipation | | Laxatives | | Outpatient | | D | | Regularly evaluate laxative use and discontinue when opioids are stopped | | - | 6 (0.2) |
| C09 | Agents acting on RA system | | ACE inhibitors, ATII antagonists | | Outpatient | | D | | Re-evaluate ACEi + ARB combination therapy | | - | 5 (0.7) |
| J01 | Antibacterials for systemic use | | Cephalosporins (1st–4th generation) | | Inpatient | | S | | For cephalosporin prophylaxis, prefer once-daily dosing where possible | | - | 6 (0.3) |
| J01 | Antibacterials for systemic use | | Various | | Outpatient | | S | | Evaluate the possibility of oral switch in OPAT. | | - | 6 (0.2) |
| L04 | Immunosuppressants | | Other immunosuppressants | | Outpatient | | S | | Switch high-dose methotrexate from subcutaneous to split-dose oral administration | | - | 5 (0.7) |
| M02 | Anti-inflammatory drugs | | NSAIDs topical | | Outpatient | | S | | Reduce use of topical NSAID gels and switch to oral therapy where possible | | - | 6 (0.4) |
| N01 | Anesthetics | | Other general anesthetics (propofol) | | Inpatient | | S | | Replace propofol 1% with propofol 2% | | - | 5 (0.7) |
| S01 | Ophthalmologicals | | Various | | Outpatient | | S | | Use multidose eye drops instead of unit-dose flacons to reduce plastic waste | | - | 6 (0.3) |

D: deprescribing intervention; S: switch to most sustainable dosage form; DI: disagreement index.

ACEi/ARB: Angiotensin-Converting Enzyme inhibitors/Angiotensin Receptor Blockers (ARBs), NSAIDs: nonsteroidal anti-inflammatory drugs, OPAT: Outpatient Parenteral Antimicrobial Therapy.

## **Supplement 7: selected (de)prescribing interventions**

| 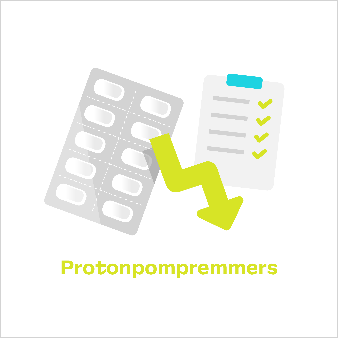 | Restrictive prescribing of **proton pump inhibitors** (as gastroprotection) | In- and outpatient setting |
| --- | --- | --- |
| 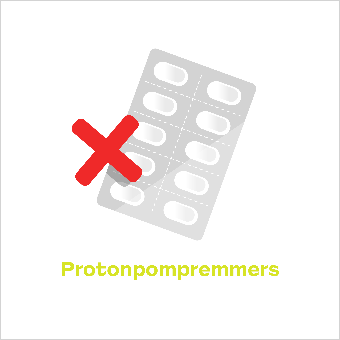 | Deprescribing **proton pump inhibitors** without a current indication | In- and outpatient setting |
| 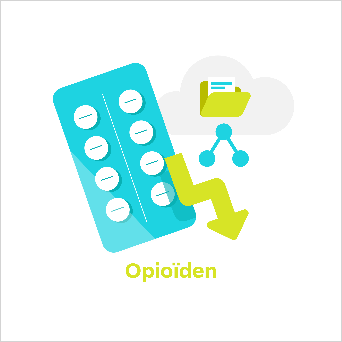 | Restrictive **opioid** prescribing with notification of indication and intended treatment duration | In- and outpatient setting |
| 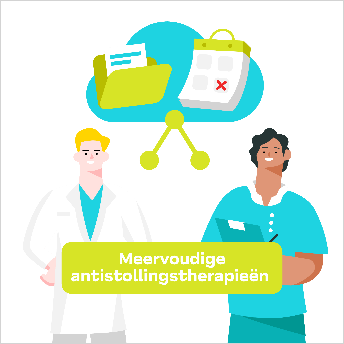 | Sharing the indication and intended treatment duration of multiple **anticoagulant therapies** | Inpatient setting |
| 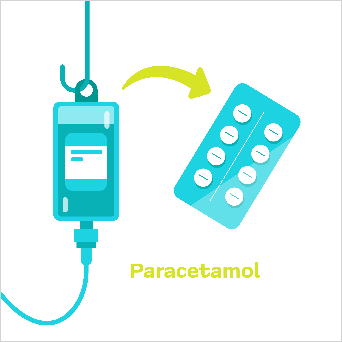 | Administering **paracetamol** orally instead of intravenously | Inpatient setting |
| 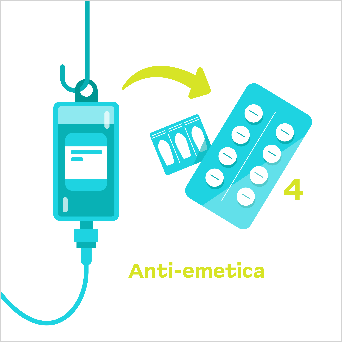 | Administering **antiemetics** enterally instead of intravenously | Inpatient setting |
| 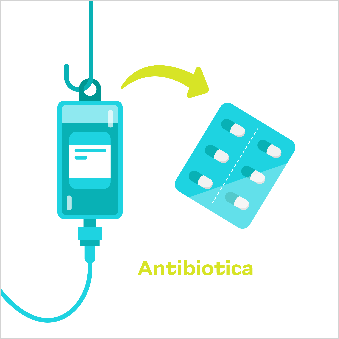 | Prescribing oral **antibiotics** when bioavailability is adequate | Inpatient setting |
| 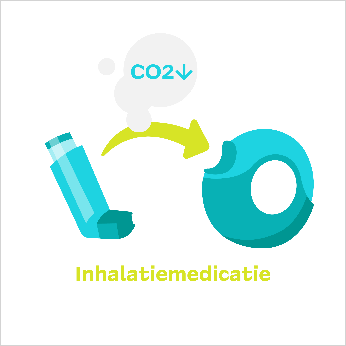 | Climate-conscious prescribing of **inhalation treatment** | Outpatient setting |
