# Supplementary material for: Advancing sustainable medication use in healthcare: a Delphi study on (de)prescribing interventions
Source: BMJ Open. 2026 May 7;16(5):e115383. doi: 10.1136/bmjopen-2025-115383 (PMC13157796; doi:10.1136/bmjopen-2025-115383)
Supplement: Supplementary file 2 [file bmjopen-2025-115383.reviewer_comments.pdf]

PEER REVIEW HISTORY

BMJ Open publishes all reviews undertaken for accepted manuscripts. Reviewers are asked to complete a checklist review form and are provided with free text boxes to elaborate on their assessment. These free text comments are reproduced below.

ARTICLE DETAILS

Title (Provisional)

Advancing sustainable medication use in health care: a Delphi-study on (de)prescribing interventions

Authors

Smale, Elisabeth Marissa; van der Giessen, Jiske Lianne; Appels, Cathelijne W.Y.; Leegwater, Emiel; Dietz, Marlieke; van den Bemt, Patricia MLA; Coenradie, Saskia; Kool, Rudolf Bertijn; Kwint, Henk-Frans; Ista, Erwin; Hunfeld, Nicole G.M.

VERSION 1 - REVIEW

**Reviewer**	**1**
**Name**	**Cateau, Damien**
**Affiliation**	**University of Geneva, Institute of Pharmaceutical Sciences of Western Switzerland**
**Date**	**27-Jan-2026**
**COI**	**None**

The manuscript is well written and adresses an important question in a relevant fashion. I have no overall reservations regarding its publication, only minor remarks and questions listed below.

Introduction

- The affirmation regarding paracetamol on lines 120-121 is not supported by reference 13, which does not mention paracetamol at all; the wrong article must have been cited.

- Line 131: it is not clear, at this point in the article, to which nation this "national program" refers.

Methods

- Line 141: why were only professionals from hospitals recruited, as the interventions considered touch both on in- and out-patient prescriptions ?

- Lines 154-6: to which time period do the data used to identifiy the top-20 medications refer? Were they the same for hospital data and insurance claims?

- Line 161: it is not clear what is meant by "possibility of modifying the prescription according to Dutch guidelines". Could you clarify?

Line 165:

- Lines 168-9: it is not clear to me if the pharmacists and physicians included in this panel were all in training; if so, why?

Results

- As the identification of relevant (de)prescribing interventions happened before the Delphi rounds, this section of the results should come before the one regarding the Delphi rounds.

- Supplement 5, table 1:

- I looked up the rationale for some of the interventions listed, as I was not familiar with them (using bolus instead of infusion for antibiotics and loop diuretics); it would be interesting to include a brief description of the rationale for each proposed intervention, for the readers' benefit.

- I did not, at first, identify that the references in this table are not the ones of the article. As it is, tere are 3 different reference lists in the manuscript (main article, protocol, supplement 5), which is confusing. Could you either include the references for table 5.1in the main reference list, or at least use reference numbers following the ones from the main reference list for this table , to avoid confusion?

Discussion

- No comments for this section

DelphiSTAR checklist:

- It is not clear how item 6 (methods consulting) is adressed at the indicated page.

**Reviewer**	**2**
**Name**	**Watson, Margaret**
**Affiliation**	**University of Strathclyde, Strathclyde Institute of Pharmacy and Biomedical Sciences**
**Date**	**06-Feb-2026**
**COI**	**None**

Thank you for the opportunity to review this manuscript which described a Delphi study that was novel in its identification and ranking of pharmaceuticals in relation to sustainability and environmental concerns. It would have been useful to have had additional information regarding the process of evidence identification and synthesis used to inform the content of the Delphi as this was the fundamental underpinning of the study and was not described in substantial detail.

The Discussion could have been developed further to reflect that only physicians and pharmacists were included in this study and that the patient/public voice was not represented/considered. This will be an important element in achieving person-centred prescribing and deprescribing decisions/actions.

It would also have been interesting and useful to have some discussion of effective strategies i.e. behaviour change techniques, that have been shown to be effective e.g. switching iv to oral more quickly - what actual techniques/interventions have achieved this - is there sufficient evidence of HOW these types of deprescribing or switching activities can be achieved effectively, sustaibly and at scale?

The additional suggestions from Round 1 were incorporated into Rounds 2 and 3 and this needs to be included in the methods.

Include a statement that items for which consensus was achieved in Round 1 were not presented in Round 2 (except for the ones that required modification).

Was any exploration/analysis undertaken to compare rankings by professional group i.e. physicians/prescribers? What additional information might have be gained from undertaking this analysis?

Provide explanation re Rank Sum of Weighting - what do higher vs lower scores mean - include in footnote to ensure ease of comprehension for readers (and reviewers).

Minor "criterium" spelling error - criterion is probably the term to be used instead.

Supplement 6 - doesn't add or provided new information to that presented in main manuscript -suggest omit.

Finally, what a wonderful incentive - planting trees - inspired and novel.

**Reviewer**	**3**
**Name**	**Jaworska, Natalia**
**Affiliation**	**University of Calgary, Critical Care Medicine**
**Date**	**12-Feb-2026**
**COI**	**None**

Remarks to the Authors:

Thank you for the opportunity to review your work.

The authors present a modified Delphi study to identify and prioritize the most appropriate prescribing and deprescribing interventions in both inpatient and outpatient hospital care. The authors' aim with this Delphi study was to determine interventions that have the potential to advance environmentally sustainable healthcare. The authors developed consensus on 42 recommendations on prescribing and deprescribing interventions for 18 medication classes. They further ranked the top 20 of those recommendations (for both inpatient and outpatient settings) as

priority for implementation that included recommendations such as changing intravenous to oral paracetamol administration, discontinuation of chronically used proton pump inhibitors without clear indication and selecting oral or intravenous antibiotics in circumstances of good oral bioavailability.

I commend the authors on the work put forth to complete this study. This is an important topic as there is increasing recognition of the impact that the delivery of healthcare impacts the environment with little current focus on environmental sustainability. The manuscript is overall clear and well-written. There are several methodological queries provided for the authors' consideration. I provide the following suggestions for strengthening the manuscript.

1. Within the introduction, the authors state "Medications for which the potential harms outweigh their therapeutic benefits are considered potentially inappropriate (8, 9). Studies showed that up to 92% of patients may receive potentially inappropriate medications, depending on the population, clinical context, and drug class (10)." Although reducing potentially inappropriate medication prescribing and use is important to reduce patient adverse events and unnecessary medication burden, it is unclear how potentially inappropriate medications relates directly to environmental sustainability. As this is the underpinning aim of the manuscript, the rational for introducing or discussing potentially inappropriate medication prescribing in the context of environmental sustainability should be strengthened.
2. I think it is important to better define what is meant by environmental sustainability in the context of medication use that the authors are aiming to address. Medication related environmental sustainability can be used in reference to several stages of the medication production, transport, dispensing and administration process. It would be important for the authors to provide their definition of environmental sustainability contextualized in the scope of this work. Otherwise, it remains unclear as to how environmental sustainability was integrated into Delphi statements.
3. How were environmental sustainability statements determined? I recognize that the authors have based their statements on the hypothesis that more frequently used medications are more likely to have greater environmental impact, however there is a lack of evidence presented to support that this hypothesis is true and that this approach has the potential to impact pollution related to medications. What is the evidence that supports this approach that medication appropriateness and deprescribing is related or impacts environmental sustainability?
4. Additional detail on how participants were to interpret and rate appropriateness would be useful. Was appropriateness in reference to prescribing or was appropriateness intended to include appropriateness in the context of environmentally sustainable actions?
5. I commend the authors on their ability to recruit and retain participants across multiple Delphi rounds. However, I wonder why only outpatient pharmacists but not outpatient physicians were included in the Delphi? With several recommendations specifically aimed to focus on the outpatient setting, this would seem to be an important participant group that does not seem to be represented in this study.
6. Although there was participant attrition across each round, the number of participants did not change from Round 2 to Round 3. Were participants allowed to re-join the Delphi if they did not participate in previous rounds? Were new participants recruit during each round? Clarification on inclusion criteria would be helpful.
7. What was the rational for only separating the recommendations for inpatients and outpatients during ranking in Round 3? Why were statements from the first two rounds considered together for both the inpatient and outpatient setting? This would seem to have the potential to lead to differential consideration of statements for agreement.

8. Table 2 is very comprehensive but difficult to follow, particularly with all ranked statements from both inpatient and outpatient setting together. Consideration for separating the table into one with inpatient and one with outpatient setting top ranked recommendations would make it easier to follow.
9. The conclusion statement: "This Delphi-study identified and prioritized (de)prescribing interventions to reduce environmental impact of healthcare" is more definitive of the impact of these interventions on reducing the environmental impact of healthcare than is supported by the methodologic approach presented where the statements for rating and ranking are based on the hypothesis that more frequently used medications are more likely to have greater environmental impact. There is potential that these interventions may have an environmental impact, but that conclusion is unclear to me when there is no specific framework provided that supports medication appropriateness has an environmental impact.

VERSION 1 - AUTHOR RESPONSE

Reviewer: 1

Mr. Damien Cateau, University of Geneva, University of Lausanne

Comments to the Author:

The manuscript is well written and addresses an important question in a relevant fashion. I have no overall reservations regarding its publication, only minor remarks and questions listed below.

We appreciate the time you took to carefully read our manuscript and we would like to thank you for your valuable contributions to our manuscript. Below we address the comments point-by-point.

Introduction

- The affirmation regarding paracetamol on lines 120-121 is not supported by reference 13, which does not mention paracetamol at all; the wrong article must have been cited.

Indeed, reference 13 was displayed two times in this sentence but only refers to the inhalers referred to in the first half of the sentences. We have now included the appropriate references for the claim on reducing the environmental impacts by administrating paracetamol orally instead of intravenously. This includes:

- 14. *Davies JF, et al. Environmental and financial impacts of perioperative paracetamol use: a multicentre international life-cycle assessment. Br J Anaesth. 2024;133(6).*
- *15. Hunfeld N, et al. The paracetamol challenge in intensive care: going green with paracetamol. Intense Care Med. 2024.*

- Line 131: it is not clear, at this point in the article, to which nation this "national program" refers.

We have included that this program has been initiated in the Netherlands to overcome this issue: "*Therefore, the national program 'Greening healthcare together' was initiated* ***in the Netherlands*** *to implement (de)prescribing interventions for improving environmental sustainability in in- and outpatient care (21)".*

Methods

- Line 141: why were only professionals from hospitals recruited, as the interventions considered touch both on in- and out-patient prescriptions?

The focus of this study was specifically on hospital care, as the aim was to identify and prioritize the most appropriate (de)prescribing interventions within both inpatient and outpatient hospital settings to advance environmentally sustainable healthcare. Limiting the scope to hospital settings was necessary to ensure a manageable and focused study, as first-line care presents different feasibility challenges for implementing sustainability measures. Additionally, this scope was aligned with the objectives of the "Greening Healthcare Together" program, which targets sustainability initiatives within hospital care.

- Lines 154-6: to which time period do the data used to identifiy the top-20 medications refer? Were they the same for hospital data and insurance claims?

The time period of the data used to identify the top-20 medications has now been included in the revised manuscript. Although the timelines differed slightly due to the different data sources (hospital data vs. insurance claims), the minimal difference does not impact the conclusions drawn from the analysis.

For inpatient care, the top-20 most frequently purchased medications were determined from data of four hospitals, including two university medical centers and two teaching hospitals, ***over the period February 2024 – February 2025****. The top-20 most frequently used medications by outpatients were identified from insurance claims* ***in 2024,*** *because the Dutch healthcare system is based on compulsory health insurance (24).*

- Line 161: it is not clear what is meant by "possibility of modifying the prescription according to Dutch guidelines". Could you clarify?

The phrase "possibility of modifying the prescription according to Dutch guidelines" refers to the guidelines provided in the Dutch 'Guideline Responsible Medication Switching" *2024*, which outlines when medications with the same active ingredient, strength, and formulation can be responsibly adjusted or substituted. These adjustments and substitutions are based on clinical equivalence, patient safety, and specific conditions under which switching is deemed appropriate (e.g., green or orange categories with safeguards). We have revised the manuscript to clarify this point.

Inclusion criteria for both inpatient and outpatient setting were: (I) containing an active pharmaceutical ingredient (API) and (II) ***the potential to adjust or switch the prescription in line with Dutch guidelines on when medications with the same active substance, strength, and formulation can be responsibly modified or substituted - taking into account clinical equivalence, patient safety, and conditions under which switching is appropriate*** *(26).*

Line 165:
- Lines 168-9: it is not clear to me if the pharmacists and physicians included in this panel were all in training; if so, why?

Pharmacists and physicians in training (residents) were included in the study population, alongside medical specialists and pharmacists with more experience. The inclusion of residents was intentional, as they represent the younger generation of healthcare professionals who will face the long-term consequences of environmental impacts in their practice.

The study population consisted of pharmacists, ***including hospital pharmacists, outpatient pharmacists and residents****, and* ***physicians, including medical specialists and residents working in either an in- or outpatient setting in Dutch hospitals****, because together they are responsible for prescribing practices for in- and outpatients.*

Results
- As the identification of relevant (de)prescribing interventions happened before the Delphi rounds, this section of the results should come before the one regarding the Delphi rounds.

We have changed the order of the sections “delphi-panel” and “identifying relevant (de)prescribing interventions” in line with your comment. Please see lines 321 – 355 of the revised manuscript.

Supplement 5, table 1: I looked up the rationale for some of the interventions listed, as I was not familiar with them (using bolus instead of infusion for antibiotics and loop diuretics); it would be interesting to include a brief description of the rationale for each proposed intervention, for the readers' benefit.

Based on feedback from another reviewer, we have removed Supplement 5, Table 1. To address this reviewer’s concern, Table 2 in the main manuscript now includes all the proposed interventions, along with the references that support them. While we did not include a detailed rationale for each intervention, we believe that providing the

references allows readers to access the underlying evidence supporting each intervention.

- I did not, at first, identify that the references in this table are not the ones of the article. As it is, tere are 3 different reference lists in the manuscript (main article, protocol, supplement 5), which is confusing. Could you either include the references for table 5.1in the main reference list, or at least use reference numbers following the ones from the main reference list for this table , to avoid confusion?

We have now moved the Table to the main manuscript and therefore the references are not displayed separately in the supplementary information anymore, in line with this comment.

Discussion
No comments for this section

DelphiSTAR checklist:
It is not clear how item 6 (methods consulting) is addressed at the indicated page.

The consulting method is now explicitly mentioned in the study design (lines 171 - 173) and the DelphiSTAR checklist has been updated with the corresponding page number.

"No consulting in regard to method took place".

Reviewer: 2

Prof. Margaret Watson, University of Strathclyde

Comments to the Author:
Thank you for the opportunity to review this manuscript which described a Delphi study that was novel in its identification and ranking of pharmaceuticals in relation to sustainability and environmental concerns. It would have been useful to have had additional information regarding the process of evidence identification and synthesis used to inform the content of the Delphi as this was the fundamental underpinning of the study and was not described in substantial detail.

Thank you for your valuable feedback that helped us improve our manuscript. We have now included more extensive details on the evidence identification that informed the input of the Delphi-study. This included an extensive literature search combined with expert opinion of a panel consisting of (hospital) pharmacists (in training), medical specialists (in training) and (de-)implementation experts. In addition to the databases

searched and the specific search terms used, we have now added the in- and exclusion criteria and the quality assessment thereof in lines 195 - 214 of the revised manuscript.

All medication classes were matched to (de)prescribing interventions and options for switching to the most sustainable dosage form. This was based on (inter)national (de)prescribing guidelines, literature, specifically through Medline, Embase and Web of Science (Supplementary Information 32), and the expert opinion of a panel consisting of (hospital) pharmacists (in training), medical specialists (in training) and (de-)implementation experts. ***Inclusion criteria were based on the intervention, context, and outcomes. Interventions included deprescribing (e.g., lower-dose prescribing, tapering, withdrawal), reducing overprescribing (e.g., targeting inappropriate medicines), dosing strategies (e.g., increased dosing intervals, dose banding), eco-directed prescribing (e.g., green pharmaceuticals, low carbon options), and sustainable dosage forms (e.g., tablets vs. liquids, oral vs. parenteral). The context included medications initiated in both inpatient and outpatient hospital care. Outcome criteria were based on dose reduction (e.g., mg or % active ingredient) or environmental impact (e.g., greenhouse gas emissions for eco-directed prescribing and sustainable dosage forms). Exclusion related to study characteristics, including reviews, commentaries, current state descriptions (e.g., waste levels, concentrations in water and qualitative research on the current state). Quality assessments were conducted by the first author, reviewed by the expert panel consisting of (hospital) pharmacists (in training), medical specialists (in training) and (de-)implementation experts. The expert panel was also invited to include additional interventions based on their knowledge and experience. The identified deprescribing interventions and options for switching to the most sustainable dosage forms per medication (class) formed the input for the Delphi-study.***

The Discussion could have been developed further to reflect that only physicians and pharmacists were included in this study and that the patient/public voice was not represented/considered. This will be an important element in achieving person-centred prescribing and deprescribing decisions/actions.

In this study, we focused on gathering insights from healthcare professionals (physicians and pharmacists), as they are directly responsible for prescribing and deprescribing medications. A previous large-scale survey among Dutch medicine-users showed that they generally value environmental sustainability over costs and ease of use, with a large inter-patient variety. While the patient/public voice was not directly included in this study, we recognize the importance of considering their preferences and values in future sustainability efforts. We have therefore included a statement on person-centered prescribing in the discussion, please see lines 519 - 528 of the revised manuscript.

Equally important is incorporating the patient perspective, which was not directly addressed in this study. Evidence from a large-scale evaluation among Dutch medicine users indicates that patients generally prioritize environmental sustainability over convenience and cost (19). However, these attitudes are heterogeneous, and many patients lack specific knowledge about the environmental impact of medicines. To foster patient-centered care that balances sustainability goals with patient needs and preferences, future studies should actively assess and integrate patient perspectives. Systematically embedding environmental sustainability into prescribing in this way can ensure interventions are acceptable and effective, ultimately contributing to both improved patient care and planetary health.

It would also have been interesting and useful to have some discussion of effective strategies i.e. behaviour change techniques, that have been shown to be effective e.g. switching iv to oral more quickly - what actual techniques/interventions have achieved this - is there sufficient evidence of HOW these types of deprescribing or switching activities can be achieved effectively, sustaibly and at scale?

Discussing effective strategies and behavior change techniques, such as those used in switching from IV to oral medication, would be valuable. However, this topic falls outside the scope of the current study, which focuses primarily on identifying and prioritizing (de)prescribing interventions for environmental sustainability. That said, we have acknowledged the importance of behavior change techniques in the *Discussion* section and highlighted this as an important area for future research. We suggest future studies to explore these techniques, with particular focus on environmental sustainability in driving these interventions (lines 511 - 528).

Another key priority is determining how to effectively implement these (de)prescribing interventions in clinical practice. Successful implementation will require close collaboration between prescribers, pharmacists, and policymakers, as well as integration into existing clinical guidelines to support green behavioral change among healthcare professionals. ***However, the factors that influence healthcare professionals' adoption of these behaviors remain unclear. Although behavioral change in deprescribing has been studied (62),the role of environmental sustainability in this context is still understudied. Future research should therefore focus on developing and evaluating strategies to achieve sustainable (de)prescribing practices at scale.***

The additional suggestions from Round 1 were incorporated into Rounds 2 and 3 and this needs to be included in the methods.

Include a statement that items for which consensus was achieved in Round 1 were not presented in Round 2 (except for the ones that required modification).

We acknowledge that it is important to further clarify these aspects of the methodology. We have now updated the Methods section to explicitly state that the additional suggestions from Round 1 were incorporated into Rounds 2 and 3 and that interventions were not retested in the second Delphi-round if consensus was reached. Please see lines 274 – 277 and 287 - 296 of the revised manuscript.

Every page contained open text space to leave remarks on specific interventions. ***In the first Delphi round, each section concluded with an open-ended question asking for suggestions on any missing interventions. These additional suggestions were then used to refine and inform the interventions tested in the subsequent rounds****.*

Suggestions for ***additional*** *(de)prescribing interventions and remarks were assessed qualitatively* ***and categorized in 'new interventions to be added', 'feedback to interventions', 'feedback to Delphi-process' and 'suggestions outside of study scope'. Two authors (ES and JG) reviewed these categories. Comments in the first two categories were clustered and used to inform subsequent Delphi-rounds.*** *Interventions with intermediate median scores, disagreement and/or qualitative feedback were re-assessed in the second Delphi-round in which median scores were fed back to participants. If the open text space contained questions, specialist justification was incorporated to address these in the subsequent Delphi-round to all participants.* ***Interventions with consensus and no qualitative feedback in the first round were not presented in the second round.***

Was any exploration/analysis undertaken to compare rankings by professional group i.e. physicians/prescribers? What additional information might have be gained from undertaking this analysis?

Thank you for this suggestion. We did not conduct an exploratory sub-analysis comparing rankings between physicians and pharmacists at first, because the study was not powered for stratification – as is stated in the limitation section (lines 496 – 498). However, based on your suggestion, we analyzed the prioritization of pharmacists (Table 1) and physicians (Table 2). The results showed largely comparable prioritization patterns between the two stakeholder groups. Because the primary objective of this study was to reach consensus across professionals jointly responsible for implementing (de)prescribing interventions, we consider the combined panel results to be most relevant to the research question. Physicians and pharmacists ultimately need to collaborate in implementing these sustainability interventions; therefore, interventions supported by only one professional group would still require acceptance from the other group to be feasible in practice. As the subgroup analysis did not provide additional

insights that would meaningfully change the interpretation of the results, we decided not to include it in the manuscript to maintain focus and conciseness.

Table 1: Prioritization of (de)prescribing interventions by pharmacists

Rank	Cat rank	Intervention	Setting	Type	RSW
1	1	Prescribe oral paracetamol instead of intravenous *(paracetamol challenge)*	i	Switch	187
2	1	Avoid routine PPI initiation in low-risk patients	i	Deprescribing	177
3	2	Initiate oral antibiotics with good bioavailability	i	Switch	172
3	1	Stop chronically used PPIs without indication	o	Deprescribing	172
5	3	Prescribe oral morphine instead of intravenous morphine	i	switch	157
6	2	Reduce opioid prescriptions at discharge by standardized pain plans	i	Deprescribing	141
7	4	Administer antibiotics as bolus injection instead of infusion	i	switch	138
7	5	Administer oral iron tablets instead of liquid iron, *every other day instead of daily.*	i	switch	138
9	2	Short-term DAPT after drug-eluting stenting in patients with ACS.	o	Deprescribing	135
9	3	Stop chronically used PPIs without indication	i	Deprescribing	135

Table 2: Prioritization of (de)prescribing interventions by physicians

Rank	Cat rank	Intervention	Setting	Type	RSW
1	1	Prescribe oral paracetamol instead of intravenous *(paracetamol challenge)*	i	Switch	262
2	2	Initiate oral antibiotics with good bioavailability	i	Switch	204
3	1	Stop chronically used PPIs without indication	o	Deprescribing	199
4	1	Stop chronically used PPIs without indication	i	Deprescribing	174
5	2	Reduce opioid prescriptions at discharge by including a stop date	i	Deprescribing	163
6	3	Reduce opioid prescriptions at discharge by standardized pain plans	i	Deprescribing	161
7	3	Administer antibiotics as bolus injection instead of infusion	i	Switch	156
8	2	Reduce opioid use by communicating intended treatment duration with primary providers	o	Deprescribing	155
9	1	Switch from metered-dose inhalers to dry powder inhalers instead where possible	o	Switch	146
10	2	Initiate dry powder inhalers instead of metered-dose inhalers where possible	o	Switch	141

Provide explanation re Rank Sum of Weighting - what do higher vs lower scores mean - include in footnote to ensure ease of comprehension for readers (and reviewers).

We have now included an extended statement on the interpretation of Rank Sum of Weighing, both in the methods (lines 307 - 319 of revised manuscript) and as a footnote to Table 3 (updated based on another reviewer comment) in line 436 of revised manuscript.

Formally, the score per intervention was calculated as: $Score = \sum_{r=1}^{N} nr * (N - r + 1)$

where r represents the assigned rank to the intervention, nr the number of participants who assigned rank r, and N is the number of interventions within the category. ***Higher Rank Sum scores indicate a stronger overall preference for the intervention, with a greater consensus among panelists. Conversely, lower Rank Sum scores suggest less agreement or a lower prioritization of the intervention.*** *Interventions with the highest scores per category were compared, resulting in a top-20 of prioritized (de)prescribing interventions for hospital care.*

RSW: rank of sum weighing, in which a higher RSW indicates greatest consensus and higher prioritization of the intervention.

Minor "criterium" spelling error - criterion is probably the term to be used instead.

We have now changed “criterium" to “criterion" throughout the manuscript.

Supplement 6 - doesn't add or provided new information to that presented in main manuscript -suggest omit.

We respectfully disagree with this suggestion, as Supplement 6 provides additional valuable information that is not included in the main manuscript. Specifically, it outlines the consensus reached for each intervention in each round, including the scores per round and the reasons for re-assessment. Due to the level of detail involved, we believe this information is better suited for the supplement rather than the main text, and therefore, we have decided to retain Supplement 6. However, we have adjusted supplement 6 by moving the main table that described all tested interventions to Table 2A and 2B of the revised manuscript.

Finally, what a wonderful incentive - planting trees - inspired and novel.

Thank you for your compliment!

Reviewer: 3 (see also attached)

Dr. Natalia Jaworska, University of Calgary

Comments to the Author:
Thank you for the opportunity to review your work.

The authors present a modified Delphi study to identify and prioritize the most appropriate prescribing and deprescribing interventions in both inpatient and outpatient hospital care. The authors' aim with this Delphi study was to determine interventions that have the potential to advance environmentally sustainable healthcare. The authors developed consensus on 42 recommendations on prescribing and deprescribing interventions for 18 medication classes. They further ranked the top 20 of those recommendations (for both inpatient and outpatient settings) as priority for implementation that included recommendations such as changing intravenous to oral paracetamol administration, discontinuation of chronically used proton pump inhibitors without clear indication and selecting oral or intravenous antibiotics in circumstances of good oral bioavailability.

I commend the authors on the work put forth to complete this study. This is an important topic as there is increasing recognition of the impact that the delivery of healthcare impacts the environment with little current focus on environmental sustainability. The manuscript is overall clear and well-written. There are several methodological queries provided for the authors' consideration. I provide the following suggestions for strengthening the manuscript.

We appreciate the time you took to carefully read our manuscript and we would like to thank you for your valuable contributions to our manuscript. Below we address the comments point-by-point.

1. Within the introduction, the authors state "Medications for which the potential harms outweigh their therapeutic benefits are considered potentially inappropriate (8, 9). Studies showed that up to 92% of patients may receive potentially inappropriate medications, depending on the population, clinical context, and drug class (10)." Although reducing potentially inappropriate medication prescribing and use is important to reduce patient adverse events and unnecessary medication burden, it is unclear how potentially inappropriate medications relates directly to environmental sustainability. As this is the underpinning aim of the manuscript, the rational for introducing or discussing potentially inappropriate medication prescribing in the context of environmental sustainability should be strengthened.

We agree that the connection between (potentially) inappropriate medications and environmental sustainability could be made clearer. It could be argued that improved patient safety reduces harm, avoiding complications that require additional treatments, ultimately reducing environmental impacts. However, this has not been clearly demonstrated through empirical research. In the manuscript, we therefore introduced the concept of deprescribing potentially inappropriate medications as a starting point for

reducing medication use, thereby minimizing the environmental impact associated with manufacturing and distributing these medications. In response to your comment, we have revised the introduction to elaborate on the rationale for linking the reduction of potentially inappropriate medication prescribing with environmental sustainability.

Optimizing medication use and reducing low-value prescriptions offer important opportunities to improve clinical outcomes and patient safety, ***while improving environmental sustainability through the reduction of the footprint associated with manufacturing and distributing these medications*** *(6, 7). Medications for which the potential harms outweigh their therapeutic benefits are considered potentially inappropriate (8, 9). Studies showed that up to 92% of patients may receive potentially inappropriate medications, depending on the population, clinical context, and drug class (10).*

2. I think it is important to better define what is meant by environmental sustainability in the context of medication use that the authors are aiming to address. Medication related environmental sustainability can be used in reference to several stages of the medication production, transport, dispensing and administration process. It would be important for the authors to provide their definition of environmental sustainability contextualized in the scope of this work. Otherwise, it remains unclear as to how environmental sustainability was integrated into Delphi statements.

Environmental sustainability has been defined in the introduction as follows: "*Sustainable prescribing therefore encompasses both clinical and environmental optimization, ensuring that patients receive the most effective and environmentally responsible treatment at the optimal dose while avoiding unnecessary or harmful medications (16)*". However, we agree with the reviewer that this definition is critical for the assessment by participants, and therefore further elaboration on the Delphi-statements and cues for evaluating and prioritizing appropriateness of the interventions was added to the revised manuscript (lines 243 - 263). The exact wording of the Delphi-questionnaires can be found in the Supplementary Information 3.

The Delphi-study consisted of three Delphi-rounds in total. ***In all communications, sustainable prescribing was defined as: "the clinical and environmental optimization of treatment to ensure that patients receive the most effective and environmentally responsible treatment at the optimal dose while avoiding unnecessary or harmful medications" in relation to deprescribing (e.g., reducing unnecessary medication use by not starting, lowering the dosage, tapering, or stopping medications) and/or switching to more sustainable administration forms (e.g., choosing the most sustainable variant of a medication, related to the route of administration and form).*** *After an introduction on study objectives, informed consent and general personal characteristics, including occupation, medical specialty and type*

of hospital of employment, participants were requested to rate interventions per setting (outpatient vs. inpatient) and intervention type (deprescribing vs. switching to the most sustainable dosage form) (Supplementary Information 3). ***Participants were requested to rate all the interventions on appropriateness for implementation in Dutch hospitals to improve environmental sustainability, with specific consideration of (I) the effectiveness and safety of the treatment remain guaranteed; (II) the environmental impact of the treatment is reduced, for example by reduced dosage and/or less packaging material; (III) the initiative can be implemented in Dutch hospitals Additionally, other factors for evaluation were pointed out, specifically the level of scientific evidence or practical experience demonstrating that the initiative is appropriate for reducing environmental impact and how well the interventions fit into existing care pathways and/or their ease of implementation.*** *All interventions were substantiated with the type of evidence including a URL to the corresponding research.*

3. How were environmental sustainability statements determined? I recognize that the authors have based their statements on the hypothesis that more frequently used medications are more likely to have greater environmental impact, however there is a lack of evidence presented to support that this hypothesis is true and that this approach has the potential to impact pollution related to medications. What is the evidence that supports this approach that medication appropriateness and deprescribing is related or impacts environmental sustainability?

We acknowledge the limited direct evidence linking medication appropriateness and deprescribing to environmental sustainability. However, our approach is based on the principle that higher medication use typically correlates with a larger environmental footprint, including resource consumption and waste. Existing research, including the Cochrane Review on Deprescribing (Gnjidic et al., 2021), supports the idea that reducing unnecessary or inappropriate medications can benefit both clinical outcomes and sustainability by lowering the pharmaceutical burden. Deprescribing strategies, such as reducing medications like antihypertensives and psychotropics, align with sustainable healthcare practices by reducing production, transportation, and disposal impacts. While we did not directly quantify the environmental impact, we based our interventions on literature indicating that reducing overprescribing and promoting appropriate medication use inherently contributes to a smaller environmental footprint. We have updated the manuscript to clarify that our focus is on reducing resource use and waste, a universally accepted approach to sustainability in healthcare.

Optimizing medication use and reducing low-value prescriptions offer important opportunities to improve clinical outcomes and patient safety, while improving environmental sustainability through the reduction of the footprint associated with manufacturing and distributing these medications (6, 7). This approach is grounded

in the understanding that reducing unnecessary medication use not only improves patient care but also minimizes the environmental impact of pharmaceuticals(8).

4. Additional detail on how participants were to interpret and rate appropriateness would be useful. Was appropriateness in reference to prescribing or was appropriateness intended to include appropriateness in the context of environmentally sustainable actions?

Based on this valuable comment, we have included a more extensive description of the framing of appropriateness and environmental sustainability in the Delphi-study in lines 243 - 263 of the revised manuscript. This information can also be found in the Supplementary Information 3 that encompasses the complete survey.

The Delphi-study consisted of three Delphi-rounds in total. ***In all communications, sustainable prescribing was defined as: "the clinical and environmental optimization of treatment to ensure that patients receive the most effective and environmentally responsible treatment at the optimal dose while avoiding unnecessary or harmful medications" in relation to deprescribing (e.g., reducing unnecessary medication use by not starting, lowering the dosage, tapering, or stopping medications) and/or switching to more sustainable administration forms (e.g., choosing the most sustainable variant of a medication, related to the route of administration and form).*** *[...]*

Participants were requested to rate all the interventions on appropriateness for implementation in Dutch hospitals to improve environmental sustainability, with specific consideration of (I) the effectiveness and safety of the treatment remain guaranteed; (II) the environmental impact of the treatment is reduced, for example by reduced dosage and/or less packaging material; (III) the initiative can be implemented in Dutch hospitals. Additionally, other factors for evaluation were pointed out, specifically the level of scientific evidence or practical experience demonstrating that the initiative is appropriate for reducing environmental impact and how well the interventions fit into existing care pathways and/or their ease of implementation. All interventions were substantiated with the type of evidence including a URL to the corresponding research.

5. I commend the authors on their ability to recruit and retain participants across multiple Delphi rounds. However, I wonder why only outpatient pharmacists but not outpatient physicians were included in the Delphi? With several recommendations specifically aimed to focus on the outpatient setting, this would seem to be an important participant group that does not seem to be represented in this study.

We have included both physicians working in inpatient and outpatient care in the Delphi panel. However, due to the overlap in roles (e.g., a physician may work in both settings, for instance inpatient one week, outpatient the next), we did not explicitly differentiate between inpatient and outpatient physicians in the questionnaire. Instead, we asked participants to provide their specialty, which is closely aligned with the frequency of their work in each setting. To clarify this we have added a statement to the methods section.

The study population consisted of pharmacists, including hospital pharmacists, outpatient pharmacists and residents, and physicians, including medical specialists and residents ***working in either an in- or outpatient setting in Dutch hospitals****, ….*

6. Although there was participant attrition across each round, the number of participants did not change from Round 2 to Round 3. Were participants allowed to re-join the Delphi if they did not participate in previous rounds? Were new participants recruit during each round? Clarification on inclusion criteria would be helpful.

Thank you for your comment. The Delphi panel was invited separately for each round. Participants were not required to have participated in previous rounds and could choose to join any specific round based on their availability. Additionally, they could re-join subsequent rounds if they had not participated earlier. We have now updated the manuscript to clarify this point.

To account for a minimum 70% response rate per round, initially 30 participants per stakeholder group (e.g., 30 physicians and 30 pharmacists) were recruited to ensure that at least 20 participants would complete all rounds of the Delphi-study (27). ***Participants were invited to each Delphi round separately, and participation in a previous round was not a requirement to join subsequent rounds.***

7. What was the rational for only separating the recommendations for inpatients and outpatients during ranking in Round 3? Why were statements from the first two rounds considered together for both the inpatient and outpatient setting? This would seem to have the potential to lead to differential consideration of statements for agreement.

This might be a misunderstanding of the manuscript, because during all Delphi-rounds the interventions were split into separate pages based on intervention type (deprescribing versus most sustainable dosage form) and care setting (in- and outpatient care). Although this was already briefly mentioned in the methods, we have now included an explicit clarification in the methods section on data collection of Delphi round 1 and 2:

The first two Delphi-rounds evaluated the appropriateness of potential (de)prescribing interventions within each medication class ***per setting (outpatient vs. inpatient) and intervention type (deprescribing vs. switching to the most sustainable dosage form)****.*

8. Table 2 is very comprehensive but difficult to follow, particularly with all ranked statements from both inpatient and outpatient setting together. Consideration for separating the table into one with inpatient and one with outpatient setting top ranked recommendations would make it easier to follow.

Based on this valuable comment we have revised Table 2. Table 2A and 2B now described the identified (de)prescribing interventions and their evaluated appropriateness for in- and outpatients respectively, while Table 3 encompassed the prioritization.

9. The conclusion statement: "This Delphi-study identified and prioritized (de)prescribing interventions to reduce environmental impact of healthcare" is more definitive of the impact of these interventions on reducing the environmental impact of healthcare than is supported by the methodologic approach presented where the statements for rating and ranking are based on the hypothesis that more frequently used medications are more likely to have greater environmental impact. There is potential that these interventions may have an environmental impact, but that conclusion is unclear to me when there is no specific framework provided that supports medication appropriateness has an environmental impact.

We agree that a more explicit framework quantifying the environmental impact of the interventions is missing, which has been highlighted as one of the key limitations of the current study (revised manuscript lines 491- 502). However, all the interventions prioritized in the study are based on the reduction of medication and material use and/or align with existing literature on minimizing environmental impact (e.g., IV-to-oral switches and inhalation medications). While the method used assumes that more frequently prescribed medications are more likely to have a greater environmental impact, it is grounded in the principle of reducing unnecessary use. Following circularity principles, reducing the number of medications, particularly inappropriate ones, not only decreases the environmental burden of production and disposal but is inherently more sustainable than using additional resources. This was also verified by the Delphi-panel who assessed the interventions for appropriateness to reduce environmental impact. We therefore believe that the conclusion of "identified and prioritized (de)prescribing interventions to reduce environmental impact" is justified by the underlying method.

VERSION 2 - REVIEW

Reviewer 1

Name Cateau, Damien

Affiliation University of Geneva, Institute of Pharmaceutical Sciences of Western Switzerland

Date 31-Mar-2026

COI

Thanks to the authors for adressing my comments, and those of the other reviewers.

I have comments for table 2A: the Therapeutic group, Medication class and Reference are missing for the intervention "Reconcile/taper dosing based on HbA1c and glucose"; references are also missing for "Prescribe enteral metoclopramide instead of intravenous" and "Prescribe oral ondansetron or granisetron instead of intravenous".

I have no further comments.

Reviewer 3

Name Jaworska, Natalia

Affiliation University of Calgary, Critical Care Medicine

Date 02-Apr-2026

COI

Thank you for the opportunity to review your work again. The authors present a modified Delphi study to identify and prioritize the most appropriate prescribing and deprescribing interventions in both inpatient and outpatient hospital care. The authors' aim with this Delphi study was to determine interventions that have the potential to advance environmentally sustainable healthcare. The responses from the reviewers were comprehensive and address concerns raised during my previous review. I have no additional comments to add to this revision.
